# Re‐evaluating Coho salmon (*Oncorhynchus kisutch*) conservation units in Canada using genomic data

**DOI:** 10.1111/eva.13489

**Published:** 2022-10-18

**Authors:** Amanda Xuereb, Quentin Rougemont, Xavier Dallaire, Jean‐Sébastien Moore, Eric Normandeau, Bérénice Bougas, Alysse Perreault‐Payette, Ben F. Koop, Ruth Withler, Terry Beacham, Louis Bernatchez

**Affiliations:** ^1^ Département de Biologie Institut de Biologie Intégrative et des Systèmes (IBIS) Université Laval Québec Québec Canada; ^2^ CEFE, Centre d'Ecologie Fonctionnelle et Evolutive, UMR 5175, CNRS, Univ Montpellier, CNRS, EPHE, IRD Univ Paul Valéry Montpellier Montpellier France; ^3^ Department of Biology University of Victoria Victoria British Columbia Canada; ^4^ Department of Fisheries and Ocean Pacific Biological Station Nanaimo British Columbia Canada

**Keywords:** conservation genomics, conservation planning, genotype–environment association, genotyping‐by‐sequencing, salmon

## Abstract

Conservation units (CUs) are important tools for supporting the implementation of standardized management practices for exploited species. Following the adoption of the Wild Salmon Policy in Canada, CUs were defined for Pacific salmon based on characteristics related to ecotype, life history and genetic variation using microsatellite markers as indirect measures of local adaptation. Genomic data sets have the potential to improve the definition of CUs by reducing variance around estimates of population genetic parameters, thereby increasing the power to detect more subtle patterns of population genetic structure and by providing an opportunity to incorporate adaptive information more directly with the identification of variants putatively under selection. We used one of the largest genomic data sets recently published for a nonmodel species, comprising 5662 individual Coho salmon (*Oncorhynchus kisutch*) from 149 sampling locations and a total of 24,542 high‐quality SNPs obtained using genotyping‐by‐sequencing and mapped to the Coho salmon reference genome to (1) evaluate the current delineation of CUs for Coho in Canada and (2) compare patterns of population structure observed using neutral and outlier loci from genotype–environment association analyses to determine whether separate CUs that capture adaptive diversity are needed. Our results reflected CU boundaries on the whole, with the majority of sampling locations managed in the same CU clustering together within genetic groups. However, additional groups that are not currently represented by CUs were also uncovered. We observed considerable overlap in the genetic clusters identified using neutral or candidate loci, indicating a general congruence in patterns of genetic variation driven by local adaptation and gene flow in this species. Consequently, we suggest that the current CU boundaries for Coho salmon are largely well‐suited for meeting the Canadian Wild Salmon Policy's objective of defining biologically distinct groups, but we highlight specific areas where CU boundaries may be refined.

## 1 INTRODUCTION

Cumulative effects of climate change, disease, habitat degradation and exploitation continue to threaten the survival and persistence of biodiversity (Díaz et al., 2019). To mitigate these effects, conservation and management strategies are needed to ensure the preservation of species diversity and the sustainability of vital resources on which humans depend (IPBES, 2019). For example, fisheries and aquaculture provide about 17% of the total animal protein consumed by more than 3 billion people, as well as jobs for nearly 60 million people worldwide (FAO, 2020). Despite the rapid decline or near collapse of many exploited fish populations (Pauly & Zeller, 2016), global consumption of fish and production from capture fisheries continue to increase (FAO, 2020). Genetic and genomic methods provide valuable information to inform conservation and management practices (Allendorf et al., 2010; Andrello et al., 2022; Flanagan et al., 2018), including numerous applications for fisheries such as identifying stocks and their spatial structure, estimating effective population sizes and managing mixed‐stock fisheries (Bernatchez et al., 2017).

One of the most common applications of genetic or genomic information for resource management and conservation is in characterising the spatial structure of populations and in turn delineating distinct conservation units (CUs; Funk et al., 2012; Waples et al., 2008). In general, CUs are discrete groups whose boundaries are defined based on differentiating criteria so as to effectively allocate management resources among units and improve the tractability of monitoring and/or reporting on the success of conservation actions (DFO, 2009). Depending on the scale and objective of conservation efforts, there are several different categories of CUs that are used to define biologically meaningful groupings. At the broadest scale, evolutionary significant units (ESUs) encompass reproductively isolated and adaptively divergent groups (e.g. populations or groups of populations) that are thus considered to be evolutionarily independent (Fraser & Bernatchez, 2001; Moritz, 1994; Waples, 1991). In Canada, the Committee on the Status of Endangered Wildlife (COSEWIC) employs a related concept to defining Designatable Units (DUs), which are defined as intraspecific units that are biologically distinct (e.g. based on genetic divergence, life history, morphological traits or habitat) and may differ in their conservation status (Green, 2005). For example, using a hierarchical approach, Mee et al. (2015) defined DUs for the purposes of prioritizing lake whitefish (*Coregonus clupeaformis*) populations in Canada based on reproductive isolation, phylogeography, local adaptation and biogeographic regions. At a smaller scale, management units (MUs) may be defined as local populations that are demographically independent and thus should be managed separately (Funk et al., 2012; Waples & Lindley, 2018). In general, ESUs and MUs may be considered as hierarchical categories of CUs, where there may be several MUs within a single ESU that are managed over relatively short timescales (e.g. monitoring/managing restocking efforts and regulating harvest allocations).

Prior to the availability of genomic data for many species, intraspecific CUs could be identified on the basis of a relatively small number of selectively neutral genetic markers, such as microsatellites. While neutral markers could be used to identify distinct groups resulting from isolation, characterising adaptive differences relied on the use of proxies such as morphology, life history or habitat. It is now possible to sequence genome‐wide genetic variation across thousands of markers for most organisms, making it feasible to generate large‐scale genomic data sets for applied conservation and management (Allendorf et al., 2010; Waples & Lindley, 2018). These larger data sets increase precision and accuracy of inferences regarding population structure and connectivity (Benestan et al., 2015; Rougemont et al., 2019; Vendrami et al., 2017), and allow for screening of genome‐wide markers to identify variants potentially underlying adaptation based on associations with environmental conditions or phenotypes (Dallaire et al., 2021; Moore et al., 2017; Rellstab et al., 2015; Thompson et al., 2020; Waples et al., 2022). Different patterns of genetic structure may be detected by either neutral or putatively adaptive genetic markers, thereby warranting consideration of different approaches for spatial management (Hanson et al., 2020; Xuereb et al., 2021). For example, Pecoraro et al. (2018) identified three distinct stocks of yellowfin tuna (*Thunnus albacares*) based on neutral loci, but a total of five stocks were ascertained based on putatively adaptive (i.e. outlier) loci. In another example, Sandoval‐Castillo et al. (2018) detected divergent genetic clusters based on adaptive variation primarily correlated with sea surface temperature and oxygen concentration in the greenlip abalone (*Haliotis laevigata*) in southern Australia, whereas neutral genetic markers identified a single metapopulation. These studies support defining separate categories of CUs that reflect different processes underlying genetic distinctiveness between groups. For example, MUs may be defined as genetically distinct groups that arise due to connectivity (or lack thereof) detected using neutral markers, while adaptive units (AUs) represent adaptively differentiated groups defined using markers that are putatively under selection, as proposed by Funk et al. (2012). For some species, distinguishing between neutral and adaptive loci may not necessarily lead to differences in CU definitions. This would be expected in biological situations where local adaptation resulting from the effect of natural selection is a major driver of population structure, for instance as documented in Atlantic salmon *Salmo salar* (Moore et al., 2014). However, other studies have demonstrated the potential need for defining distinct MUs and AUs to support genetic‐based conservation and management objectives for various taxa (e.g. Barbosa et al., 2018; Silva et al., 2020).

**FIGURE 1:**
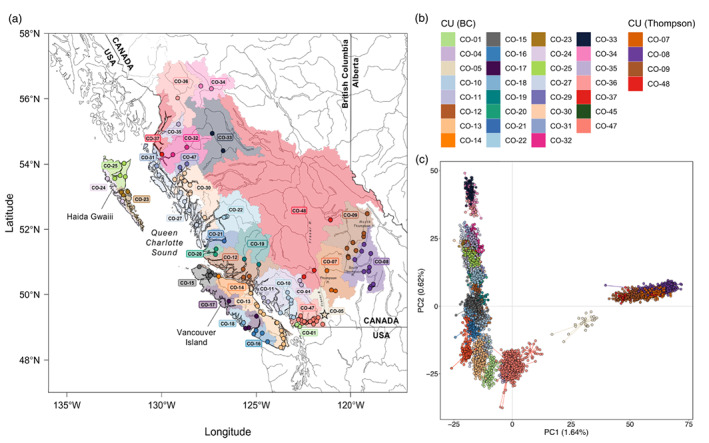
(a) Map of sampling locations (coloured points). Each point is coloured according to the CU within which it is currently managed. The shaded areas delineate the boundaries of the CUs from which samples were collected. One sampling location (KLU; CO‐45; coordinates: 60.11583, −137.0361) has been omitted from the map for ease of viewing the locations of the majority of sampling locations. The location of KAW is indicated by the star; (b) colour scheme used to represent CU designation on the map (a) and across all figures in which colour corresponds to CU; (c) PCA on individual genotypes. Points are coloured according to their currently designated CU using the colour scheme in (b). CU, conservation units; PCA, principal components analysis.

Pacific salmon, all of which are semelparous anadromous species and which include Coho (*Oncorhynchus kisutch*), Chum (*O. keta*), Chinook (*O. tshawytscha*), Sockeye (*O. nerka*) and Pink (*O. gorbuscha*) salmon, are an important social, cultural and economic resource in Canada (DFO, 2005). However, declines in Coho salmon abundance have been observed for the last five decades, and many native populations have disappeared from parts of their range (Bendriem et al., 2019; Gustafson et al., 2007; Irvine & Fukuwaka, 2011), leading to massive reductions in catch value and commercial fishery closures (Beamish et al., 1999). Despite efforts to mitigate the loss of threatened populations, for example through harvest restrictions and/or fishery closures, the drastic reduction in population size has yet to be reversed. As such, the establishment and continued evaluation of CUs to facilitate management and monitoring is a priority in Canada. In British Columbia (BC), CUs for all five species of Pacific salmon are currently defined under the Wild Salmon Policy (WSP) as distinct groups of wild salmon that are ‘sufficiently isolated from other groups’ (DFO, 2005, 2009). The framework implemented to define CUs was largely based on three primary characteristics: ecotype, life history or phenotypic variation (e.g. variation in spawn timing), and genetic distinctiveness, as measures of ecological specialisation or local adaptation (DFO, 2009). For Coho, this approach identified 43 distinct CUs in BC (Holtby & Ciruna, 2007), which were later modified to the current number of 44 CUs (Wade et al., 2019; Figure 1, see also https://axuereb.shinyapps.io/CohoSamplingMapCU/).

Several factors may contribute to the establishment of local adaptation in Coho salmon populations, implying a potential need for the definition of AUs. First, natal homing behaviour exhibited by Coho and other salmonids (Hendry et al., 2004; Quinn, 2018) is likely to maintain adaptive differences among populations. Furthermore, Coho salmon occur across a range of environmental conditions that may promote the development of locally adaptive differences due to spatially varying selection pressures. In particular, conditions affecting migration have been identified as key selective factors for anadromous salmonid species (Koch & Narum, 2020; Moore et al., 2017), including Coho salmon (Rougemont et al., 2022), which may promote the development of locally adaptive differences in phenotypes associated with spawning migration, and poses important questions regarding the configuration of CUs based on information from loci underlying local adaptations (Waples et al., 2022).

In this paper, we take advantage of the availability of a large genomic data set, with more than 24,000 markers genotyped in over 5000 individual samples, to evaluate the current definition of CUs for Coho salmon in BC. This data set was previously generated and used by Rougemont et al. (2022) to investigate patterns of population genetic structure and adaptation across the entire North American distribution of Coho salmon, from Central California up to Alaska as well as Russia. This previous work identified key environmental drivers of local adaptation, including migratory distance, temperature and precipitation, but did not aim to address applied evolutionary issues pertaining to Coho salmon conservation and management per se. Accordingly, we address outstanding questions related to conservation within Canada in this study, with the specific objectives of (a) confirming CU boundaries by comparing the current groupings of sampling locations within CUs with genetic clusters identified using a more powerful data set, and (b) determining whether genetic clusters (and correspondingly, CU definitions) differ depending on whether groups are defined using neutral or (putatively) adaptive markers. The results of this study have implications for the potential revision of CU boundaries and can help inform decisions regarding the definition of MUs and AUs for Coho salmon.

## 2 MATERIALS AND METHODS

### 2.1 Sampling and genotyping by sequencing

Samples were collected from 5662 individual Coho salmon from 149 locations in BC, Canada (Figure 1; see https://axuereb.shinyapps.io/CohoSamplingMapCU/). These sampling sites were located within 35 of the 44 established CUs (Table S1). DNA was extracted from all individuals, and libraries were prepared for genotyping by sequencing (GBS) following the protocol described in Moore et al. (2017) and Rougemont et al. (2020). Libraries were sequenced on the Ion Proton P1v2 chip (Université Laval), and raw sequencing reads were processed using the Stacks v2 pipeline (Catchen et al., 2013) with the updated Coho salmon reference genome (https://www.ncbi.nlm.nih.gov/assembly/GCF_002021735.2/), as detailed in Rougemont et al. (2020). We excluded loci that were not present in at least 60% of all populations and in 60% of individuals within each population, and loci for which the rare allele was not present in at least five samples. We kept one single‐nucleotide polymorphism (SNP) per RAD locus (i.e. the one with the highest minor allele frequency [MAF]) and removed duplicated, diverged and low‐confidence loci with the HD plot approach based on McKinney et al. (2017) and implemented in stacks_workflow (https://github.com/enormandeau/stacks_workflow). SNPs with a read depth >10 and <120 (to remove potential PCR duplicates and paralogs), with a minor allele count (MAC) >15 (which translates to a MAF threshold of ~0.15%), and that were not missing in at least 95% of the data set were retained. We filtered on MAC rather than MAF because of the large size of the data set, following Rougemont et al. (2022), since with a MAF <0.01, a SNP must be polymorphic in 56 individuals to be retained, which is larger than the sample size of nearly all of the sampled populations. Given the high levels of drift in some populations, this MAF threshold may remove many population‐specific SNPs. Therefore, the MAC approach balances the removal of biologically meaningful variants versus low‐frequency variants. We also filtered individuals with a minimum genotyping rate of 80% across all loci.

### 2.2 Population genetic structure

Analyses of broadscale population structure were performed on the full set of SNP markers. We first performed a principal components analysis (PCA) using the R package ade4 (Dray & Dufour, 2007). We also estimated individual ancestry coefficients with the program sNMF implemented in the R package LEA v2.0 (Frichot & François, 2015). We tested *K* = 1–40 clusters with 10 iterations per *K*. The optimal number of clusters was chosen using the cross‐entropy criterion based on cross‐validation, where lower cross‐entropy values indicate a better predictive model (Frichot et al., 2014).

We used a hierarchical analysis of molecular variance (AMOVA) to estimate the components of variance explained by genetic differentiation among regions (Φ_CT_) and among populations within regions (Φ_SC_), as well as among CUs (Φ_CT_) and populations within CUs (Φ_SC_). We used the *pegas* implementation of AMOVA (Paradis, 2010) in the poppr v2.9.1 R package (Kamvar et al., 2014) and tested the significance of variance components with 999 permutations. We also computed Weir and Cockerham pairwise *F*_ST_ estimator between sampling locations and 95% confidence intervals with 500 bootstrapped permutations using the StAMPP v1.6.3 package (Pembleton et al., 2013) in R and computed expected heterozygosity (H_s_) in each sampled population using adegenet v2.1.3 (Jombart & Ahmed, 2011).

Previous studies identified a strong signal of latitudinal isolation by distance (IBD) across the entire North American distribution of Coho salmon due to the northward postglacial population expansion (Rougemont et al., 2020, 2022). Similarly, we evaluated the relationship between genetic diversity (H_s_) and genetic differentiation (*β*_ST_) and the distance from the southernmost site to assess the potential confounding effect of latitude on subsequent genotype–environment association (GEA) analyses.

### 2.3 Genotype–environment associations

Environmental data were extracted from the WorldClim v2.0 database (Fick & Hijmans, 2017) from within the catchment areas upstream of each sampling location using ArcGIS v10.4 (ESRI, 2011). We calculated the mean, minimum, maximum, range and standard deviations (*SD*) of 19 bioclimatic variables related to temperature (°C) and precipitation (mm) between the years 1970 and 2000 (Table S2). We performed a PCA using the R package ade4 (Dray & Dufour, 2007) to reduce the full set of bioclimatic variables to a set of uncorrelated variables, separately for temperature and precipitation, and retained the significant PC axes for subsequent analyses. We also included a geological variable (four categories of rock type: 1—Metamorphic, 2—Plutonic, 3—Sedimentary and 4—Volcanic; Table S2), obtained from the USGS database (Garrity & Soller, 2009) and a variable related to migration harshness, which was computed as the product of river length and elevation standardised to a mean of 0 and SD of 1, hereafter referred to as ‘normalized distance’ (see Moore et al., 2017; Rougemont et al., 2022). We then used two GEA approaches to detect candidate outlier SNPs correlated with environmental variables: (1) Redundancy analysis (RDA) and (2) Latent factor mixed models (LFMM). While Rougemont et al. (2022) performed GEA analyses on the entire geographical range, we re‐analysed the data for the Canadian range only since signals of GEAs may differ depending on the spatial scale considered.

#### 2.3.1 Redundancy analysis

To identify candidate loci associated with environmental variables, we performed a RDA using the R package vegan v2.5.6 (Oksanen et al., 2019). This approach detects GEAs based on the covariation of groups of loci in response to multiple environmental variables, is well‐suited to detecting relatively weak molecular signatures resulting from multilocus selection and has been shown to reduce Type I errors compared with other GEA methods (Capblancq & Forester, 2021; Forester et al., 2018). Given the strong signal of IBD along the south–north axis in BC (see Section 3.1), we used the same approach as in Rougemont et al. (2022) to control for the potential confounding effect of geography in GEAs by including latitude as a conditioning variable in the model (see also Meirmans, 2012). To minimise the effects of multicollinearity among predictors, we retained environmental variables that displayed a variance inflation factor (VIF) > 10. Significance of the global RDA and of each RDA axis was assessed using an ANOVA with 1000 permutations. Candidate SNPs were then detected based on a threshold of ±3 *SD* from the mean loading on each significant RDA axis, following the approach of Forester et al. (2018).

#### 2.3.2 Latent factor mixed models

In addition to RDA, we used a LFMM approach to identify candidate loci based on GEAs (Frichot et al., 2013). Using this approach, a PCA is performed first to identify the *K* components that summarize population genetic structure (i.e. latent factors). The number of latent factors to retain was chosen using a scree plot. Then, we used the *lfmm_ridge* function in the lfmm v1.5 R package (Caye et al., 2019) to build the model, with all environmental predictor variables as fixed effects and the *K* latent factors as random effects. Latitude was also included as a predictor variable, and all outliers associated with it were excluded. We calibrated *p*‐values using the genomic control method implemented in the lfmm package and corrected for multiple testing using the Benjamini–Hochberg (or false discovery rate) method (Benjamini & Hochberg, 1995). SNPs were considered to be candidates if corrected *p*‐values were <0.01.

### 2.4 Comparison of genetic groups defined using GEA outliers and neutral markers

After identifying GEAs, we generated a putatively neutral data set and a data set consisting of candidate, or outlier, loci for each of the regional groups, to define genetic groups based on different marker types. For the neutral data set, we excluded all GEA outliers (i.e. all unique candidate SNPs detected by either RDA or LFMM). For the candidate SNP data set, we used two subsets of GEA outliers: (1) combined unique outlier SNPs detected by both RDA and LFMM and (2) only outlier SNPs detected by RDA, according to recommendations by Forester et al. (2018).

We evaluated the extent to which genetic clusters differed if defined using neutral loci or candidate loci under selection, indicating the delineation of distinct management (i.e. neutral) and AUs. We used two approaches to assess neutral and putatively adaptive genetic groups: (1) distance‐based clustering and (2) discriminant analysis of principal components (DAPC).

#### 2.4.1 Neighbour‐joining clustering

Distance‐based cluster analyses were performed to group sampling locations based on the genetic distances between all pairs of sites and to assess whether CU boundaries correspond with genetic clusters. Pairwise genetic distances between sampling locations were calculated using Cavalli‐Sforza and Edward's chord distance. Unrooted neighbour‐joining trees based on pairwise chord distance were generated using the ape v.5.5 package (Paradis & Schliep, 2019). This was the same method used to define the genetic clusters based on microsatellite loci that were used for the original definition of CUs for Pacific salmon (Holtby & Ciruna, 2007), allowing for a direct comparison with previous analyses. Node support was estimated using 1000 bootstrapped replicates with the poppr v.2.8.3 (Kamvar et al., 2014) R package. We computed cophenetic distances between all sampling locations (i.e. the distance between sampling locations on the dendrogram). We then estimated the correlation between cophenetic distance matrices computed with neutral SNPs and those computed with outlier SNPs using a Mantel test with 999 permutations, as an estimate of the congruence of trees built with different marker types. A *p*‐value <0.05 suggests that the correlation is stronger than expected based on randomized matrices.

#### 2.4.2 Discriminant analysis of principal components

Discriminant analysis of principal components is a model‐free clustering approach that aims at maximizing between‐group discrimination while minimizing within‐group discrimination (Jombart et al., 2010). We performed DAPC with prior group assignments to sampling location using the adegenet v.2.1.3 (Jombart & Ahmed, 2011) package in R. The number of PCs retained for the DAPC was chosen based on the alpha score (using the *optim.a.score* function in adegenet). We then visualized the separation of groups based on a linear discriminant analysis, which aims at maximizing variation between groups while minimizing variation within groups.

## 3 RESULTS

### 3.1 Genotyping by sequencing

A total of 24,542 SNPs were retained after filtering. Four sampling locations (Black Creek, Raft, Street and Tranquil) were excluded from further analyses due to low sample size (*n* < 10) after filtering individuals for missing data (excluding individuals missing >20% of all SNPs). In total, our final data set included 5581 samples from 146 locations across 35 CUs (Figure 1a,b). After filtering, the level of missing data was low overall, with a minimum genotype call rate of 89% within sampling locations (average proportion of non‐missing genotypes across all sampling locations = 0.97).

### 3.2 Population genetic structure

A PCA on individual genotypes from across all sampling locations revealed a split between the Thompson River (including the upper Fraser) and the rest of BC (Figure 1c). These two groups have been shown to be highly divergent in previous studies on Coho salmon population genetics and demography across its North American range (Rougemont et al., 2020, 2022). Moreover, Kawkawa Creek [KAW], which is located in the Fraser River canyon (currently in CU CO‐5), does not group with either the Thompson River or the BC, but rather shows an intermediate position between the lower Thompson River and sites in the lower Fraser River. Expected heterozygosity was lower overall in populations in the Thompson group than in populations in the BC group (Figure S1). In particular, H_s_ was lowest in Salmon River [SAL] in the South Thompson CU (CO‐08).

**FIGURE 2:**
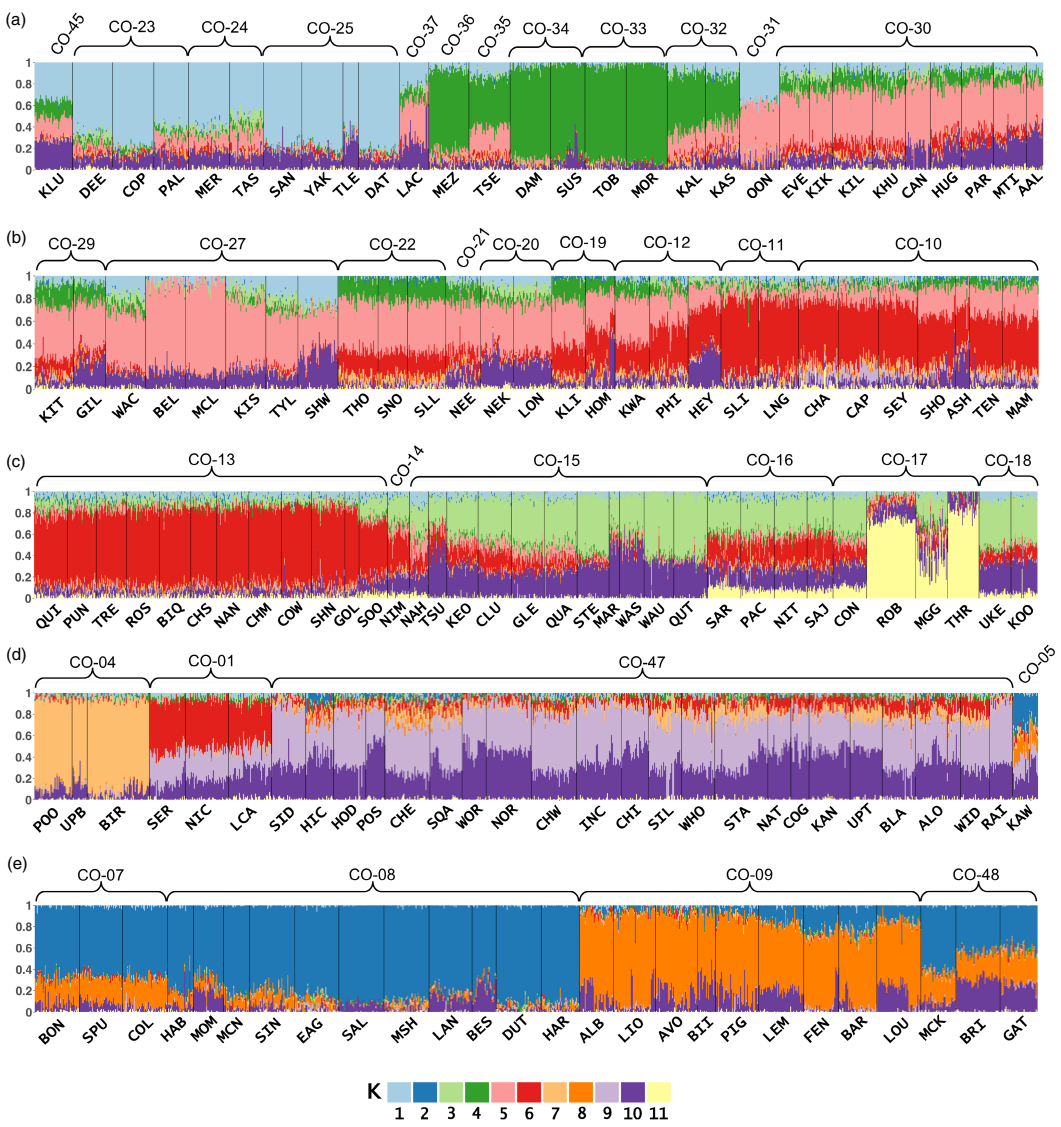
Admixture coefficients for all individuals grouped by sampling location (*x*‐axis) in BC (a–d) and Thompson (e) in *K* = 11 genetic clusters. The current conservation unit designation for each sampling location is indicated by the curly brackets above the plots.

With *K* = 2 clusters, ancestry coefficients clearly indicate a split between sampling locations in the Thompson River basin from all other sampling locations in BC, with KAW exhibiting a high degree of admixture between the two major regional clusters (Figure S2). Based on an AMOVA with two levels (Region/Population), differentiation between the two major groups (Thompson River and BC) accounted for 13.36% of the total genetic variation (Φ_CT_ = 0.134, *p* < 0.001), while 8.6% of variation was found among populations within regions (Φ_SC_ = 0.099, *p* < 0.001; Table 1). We also evaluated ancestry coefficients at *K* = 11 clusters, based on the cross‐entropy criterion (Figure S2), which showed that individuals from sampling locations within a given CU generally belonged to the same genetic group, with some exceptions (Figure 2). For example, in CO‐17, Coho from Robertson [ROB] and Thornton [THR] have a high degree of ancestry to a different genetic cluster compared with individuals from Conuma [CON] and Maggie [MGG].

**TABLE 1:** Results of an analysis of molecular variance (AMOVA) estimating variance components explained by differentiation between regional groups and between populations within regions, as well as between CUs and between populations within CUs for each regional group independently (BC and Thompson).

	*df*	Sigma	%Variance	*p*‐Value	Φ‐statistic
Between regions (BC and Thompson)	1	146.038	13.36	<0.001	0.134 (Φ_CT_)
Between populations within regions	144	93.999	8.60	<0.001	0.099 (Φ_SC_)
Error	5435	852.744	78.03		
BC region					
Between CUs	29	56.614	5.69	<0.001	0.0569 (Φ_CT_)
Between populations within CUs	89	49.382	4.97	<0.001	0.0526 (Φ_SC_)
Error	4436	888.560	89.34		
Thompson region					
Between CUs	3	27.568	5.04	<0.001	0.0504 (Φ_CT_)
Between populations within CUs	22	20.966	3.83	<0.001	0.0403 (Φ_SC_)
Error	974	498.621	91.13		

Given the strong differentiation of the Thompson River populations from other BC populations and the high degree of admixture in KAW, subsequent analyses were performed within the two regional groups separately (hereafter referred to as BC and Thompson) and excluding KAW. As a result, the BC data set consisted of 4555 individuals across 119 sampling locations in 31 CUs with a total of 24,109 SNPs. The Thompson data set consisted of 1000 individuals in 26 sampling locations within four CUs and 10,009 SNPs.

Based on an AMOVA, differentiation between CUs within the BC regional group explained 5.69% of variance (Φ_CT_ = 0.0569, *p* < 0.001) and differentiation between populations within CUs explained 4.97% of variance (Φ_SC_ = 0.0526, *p* < 0.001; Table 1). In the Thompson River group, differentiation between CUs accounted for 5.04% of the variance (Φ_CT_ = 0.0504, *p* < 0.001), while differentiation between populations within CUs accounted for 3.83% of the variance (Φ_SC_ = 0.0403, *p* < 0.001; Table 1). Pairwise *F*_ST_ values between all populations within the two regional groups were significant, except for between Poole Creek [POO] and Upper Birkenhead [UPB] (CO‐04; BC group), with mean *F*_ST_ = 0.0551 in BC (range = 0.0005–0.1637) and mean *F*_ST_ = 0.0402 in Thompson (range = 0.0035–0.0943; Tables S3 and S4). Taken together, these results confirm that CUs represent genetically significant population groupings, but also that there is substantial genetic differentiation among populations within each CU.

As Rougemont et al. (2022) reported at the scale of the entire North American distribution range, we identified a strong effect of latitudinal IBD in the BC group, as indicated by a linear decrease in genetic diversity (H_s_) and a linear increase in genetic differentiation (*β*_ST_) with increasing distance from the southernmost site (H_s_: *R* ^2^ = 0.48, *p* < 0.001; *β*_ST_: *R* ^2^ = 0.47, *p* < 0.001; Figure S3). We did not detect an effect of latitudinal IBD in the Thompson group (H_s_: *R* ^2^ = 0.003, *p* = 0.31; *β*_ST_: *R* ^2^ = −0.001, *p* = 0.34; Figure S3).

### 3.3 Genotype–environment associations

**FIGURE 3:**
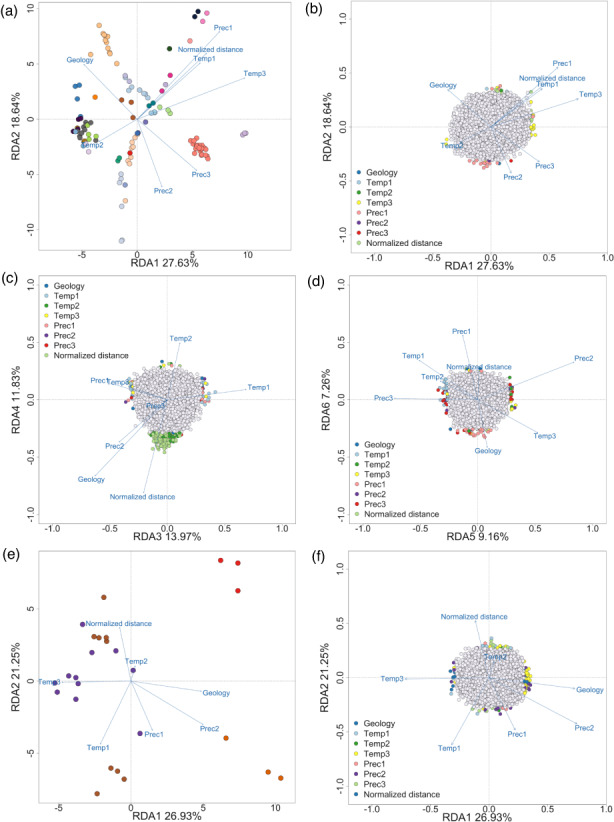
RDA results showing (a) site loadings on the first two axes (points represent sampling locations coloured according to the CU in which they are currently managed, blue arrows indicate the environmental variables) and (b–d) magnified to show SNP loadings on the first six axes in BC (coloured points represent outlier SNPs, with the colour defined according to the predictor variable with which each outlier SNP was associated, grey points represent non‐outlier SNPs); RDA results showing (e) site loadings on the first two axes and (f) magnified to show SNP loadings on the first two axes in Thompson. CU, conservation units; PrecipitationPC1, PrecipitationPC2, and PrecipitationPC3, correspond to principal components axes on precipitation variables; RDA, redundancy analysis; SNP, single‐nucleotide polymorphism; TemperaturePC1, TemperaturePC2, and TemperaturePC3, correspond to principal components axes on temperature variables.

The first three axes of the PCAs on the temperature and precipitation variables at sampling locations within the BC group were significant and cumulatively explained 85.3% and 88.9% of the total variation, respectively (Figure S4). The full RDA model included eight predictor variables: geology (rock type), normalized distance, three temperature PC axes and three precipitation PC axes. All predictor variables displayed a VIF < 10 and were thus retained. The full model was significant (*F*_8,109_ = 2.30, *p* = 0.001, adjusted *R* ^2^ = 0.078), and all predictor variables significantly contributed to the model (ANOVA, *p* < 0.05; Table S5). The first five RDA axes were significant (ANOVA, *p* < 0.05), and RDA6 was marginally significant (ANOVA, *p* = 0.06) so we retained the first six axes (Figures 3a and S5), which cumulatively captured 88% of the total explained genetic variance (Table S5). A total of 450 outlier SNPs were detected across all six axes: 141 were associated with temperature, 100 were associated with precipitation, 198 were associated with normalized migration distance and 11 were associated with geology (Figure 3b–d). With LFMM, 796 outlier SNPs were identified (corrected *p*‐value < 0.01), of which 375 were associated with temperature, 266 were associated with precipitation and 155 were associated with normalized distance. LFMM did not detect any outliers associated with geology. In total, 1117 unique outlier SNPs were detected by RDA and LFMM, and 129 were shared between both methods.

For the Thompson region, the first three axes of a PCA on temperature variables and the first two axes of a PCA on precipitation variables were significant, capturing 80% and 82% of the explained variation, respectively (Figure S6). These five PC axes were retained as predictor variables in a RDA along with geology and normalized distance, as described above. Since there is not a strong effect of latitudinal IBD as observed in the BC region, we used a principal component approach to correct for population structure when detecting candidate outlier SNPs using RDA (Capblancq & Forester, 2021). To do this, we first performed a PCA on the genetic data (allele frequencies) in populations within the Thompson group and retained the first PC axis, which was the only significant axis (*p* < 0.05) and explained 28.6% of the total genetic variation. We then incorporated this PC axis as a conditioning variable in a partial RDA (pRDA). All predictor variables, including the conditioning variable, had a VIF < 10, indicating no multicollinearity among predictors. The pRDA was significant (*F*_7,17_ = 1.3306, *p* = 0.002, adjusted *R* ^2^ = 0.073). The first two axes were significant (ANOVA, *p* < 0.05, Table S5) and were retained for detecting candidate SNPs associated with environmental predictors (Figure 3e). Given the small number of outlier SNPs detected using a threshold of ±3 *SD*s from the mean loading (only three outliers detected), we lowered the threshold to ±2.5 *SD* to increase the number of candidate loci for subsequent clustering analyses (see below). With this threshold, the RDA yielded 106 candidate SNPs associated with environmental predictors on the first two axes: 50 were associated with temperature, 29 with precipitation, 12 with normalized distance and 15 with geology (Figure 3f). Previous work testing multiple cut‐off values showed that lowering the threshold to ±2.5 *SD* had only a minor effect on the false positive rate compared with a threshold of ±3 *SD*, while also elevating true positive detections, including loci that may be under weaker selection (Forester et al., 2018). As such, the actual threshold value may be adjusted depending on the tolerance for true positive versus false positive detections, and similar thresholds have been used in other landscape genomic studies (e.g. Dorant et al., 2022). LFMM detected 18 outliers in total with a corrected *p*‐value threshold lowered to *p* < 0.05 (12 associated with normalized distance and six associated with temperature), none of which overlapped with the SNPs detected by RDA (124 unique outliers with both methods).

### 3.4 Comparison of genetic groups defined using neutral and outlier SNPs

All GEA candidate SNPs were excluded, resulting in a neutral data set consisting of 22,992 SNPs for the BC group and 9885 SNPs for the Thompson group. For the sampling locations in BC, the outlier data sets consisted of (a) 1117 unique SNPs detected by either RDA or LFMM and (b) 450 outliers detected by RDA. In the Thompson group, we performed analyses on two outlier data sets consisting of (a) 124 unique SNPs detected by either RDA or LFMM and (b) 106 SNPs detected by RDA.

**FIGURE 4:**
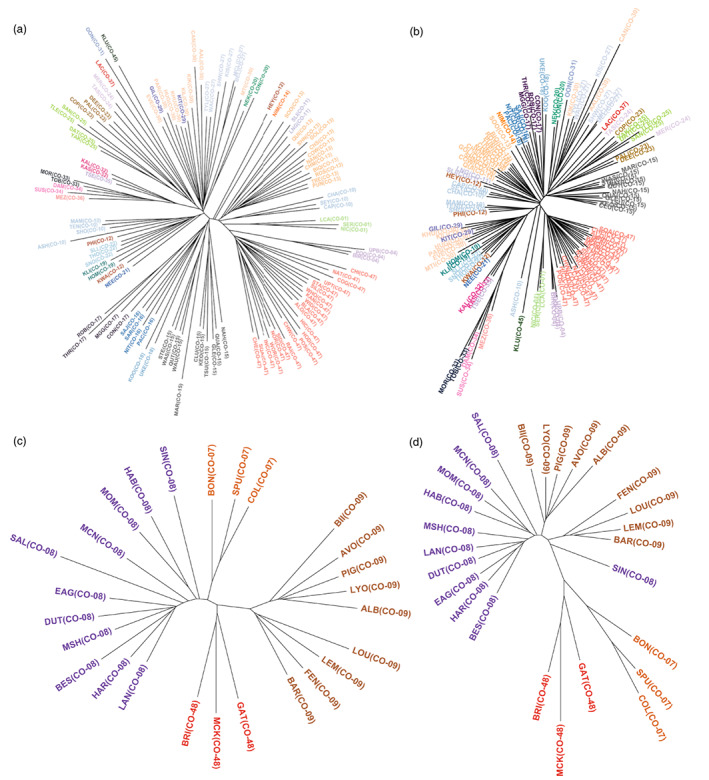
Unrooted neighbour‐joining trees based on Cavalli‐Sforza and Edward's chord distance for the BC (a, b) and Thompson (c, d) regions using neutral SNPs only (a, c) and candidate SNPs identified using GEAs (b, d). Tips correspond to sampling locations and are coloured according to the CU they currently belong to. CU, conservation units; GEA, genotype–environment association; SNP, single‐nucleotide polymorphism.

**FIGURE 5:**
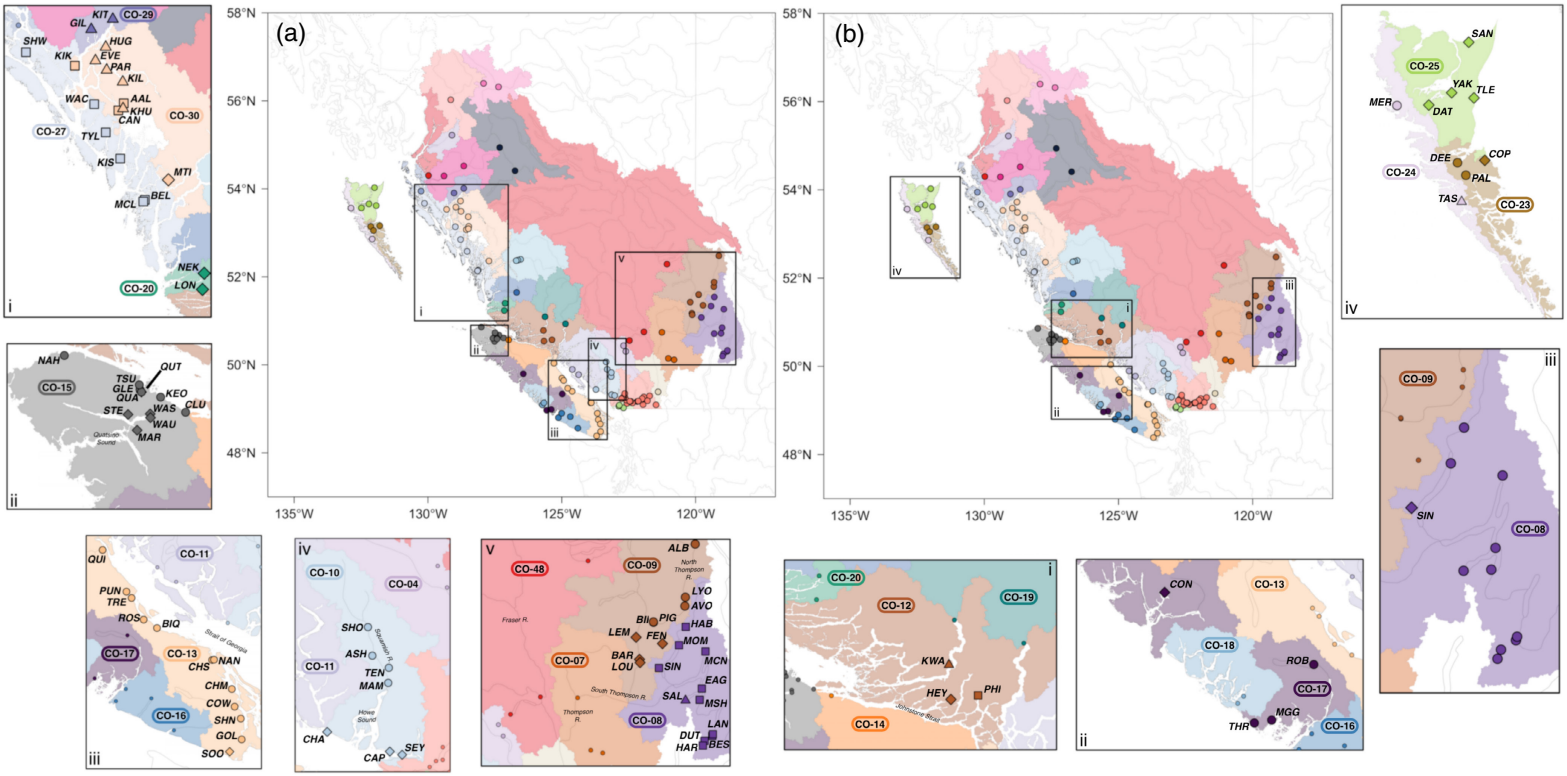
Maps showing regions (inset maps) in which inconsistences between genetic groupings and current CU boundaries were detected using (a) neutral SNPs and (b) outlier SNPs (when different from patterns based on neutral SNPs). Points represent sampling locations and are coloured according to the CU to which they are designated; the shape reflects genetic clusters, where different shapes within a CU denote a split in populations currently defined in the same CU and similar shapes across CU boundaries denote populations that are managed under separate CUs are genetically similar. The smaller points are populations that are located within the map extent but are not included in the highlighted genetic clusters. See text and Figure S8 for cluster support. CU, conservation units; SNP, single‐nucleotide polymorphism.

Using the neutral SNPs, the neighbour‐joining tree showed geographic clustering of populations within CUs in both regions (Figure 4a,c). In BC, most groups of sampling locations that are currently managed in the same CU clustered together; excluding seven CUs with only one sampling location (CO‐14, CO‐21, CO‐31, CO‐35, CO‐36, CO‐37 and CO‐45), populations in 18 of the 30 sampled CUs clustered unambiguously within their designated CU, while the remaining five CUs displayed some inconsistencies or further subdivisions compared with current delimitations (Figure 4a; see Figure S8a for cluster support). For example, in Howe Sound‐Burrard Inlet (CO‐10), three sampling locations (Seymour [SEY], Capilano [CAP] and Chapman [CHA]) formed a distinct genetic cluster from the other four sampling locations that are assigned to the same CU (Ashlu [ASH], Tenderfoot Creek [TEN], Mamquam [MAM] and Shovelnose [SHO]; Figures 4a and 5a), and ASH was also excluded from a cluster containing TEN, MAM and SHO. Two highly supported (bootstrap support > 80%) clusters were also observed in the Nahwitti Lowland CU (CO‐15), with Marble Creek [MAR] showing relatively strong dissimilarity to both clusters (Figures 4a and 5a). For the three populations sampled in CO‐12 (Southern Coastal Streams‐Queen Charlotte Strait‐Johnstone Strait‐Southern Fjords), Kwalate [KWA] and Philips [PHI] were genetically similar, but Heydon [HEY] was markedly distinct. In CO‐13 (East Vancouver Island‐Georgia Strait), Sooke [SOO] separated on a unique branch from all other populations and the cluster excluding this site was strongly supported (Figures 4a and 5a). Populations in the Northern Coastal Streams CU (CO‐30) also showed notable differentiation: Martin [MTI] was more similar to CO‐20 populations (Nekite [NEK] and Long Lake [LON]), while Canoona River [CAN], Kiskosh Creek [KIK] and Aaltanhash River [AAL] were more similar to populations in Hecate Strait Mainland (CO‐27) than to the remaining CO‐30 populations, which are genetically similar to Gilttoyees Creek [GIL] and Kitimat River [KIT] in CO‐29 (Douglas Channel‐Kitimat Arm; Figures 4a and 5a), albeit with uncertain support for the exact placement of these populations within genetic clusters (Figure S8a).

Similar patterns were observed when using all GEA candidate SNPs (Figure 4b) and with RDA candidate SNPs (Figure S7a). The cophenetic correlations between trees built using neutral and all GEA candidate SNPs, as well as RDA candidate SNPs, were significant (neutral vs. GEA: *r* = 0.56, *p* = 0.001; neutral vs. RDA: *r* = 0.57, *p* = 0.001), indicating concordance among trees built with different marker types. Altogether, populations in 15 of the 18 CUs that clearly grouped together within their designated CUs showed the same unambiguous clustering with GEA outlier loci. However, we detected additional subdivisions among populations within CUs that were not observed with neutral loci (see Figure S8b,c for cluster support with GEA and RDA outliers). For example, all four sampled populations in West Vancouver Island (CO‐17; Conuma [CON], Robertson [ROB], Thornton [THR], and Maggie [MGG]) form a strongly supported cluster with neutral markers but the exclusion of CON from this group had higher support when GEA and RDA outliers were used (Figures 4b and 5b). All three sampled populations in the Southern Coastal Streams‐Queen Charlotte Strait‐Johnstone Strait‐Southern Fjords CU (CO‐12; HEY, PHI, KWA) diverged into separate clusters when outlier loci were used (Figures 4b and 5b). Moreover, in Haida Gwaii East (CO‐23), Copper [COP] was distinct from Deena [DEE] and Pallant [PAL] and a genetic cluster containing COP and populations in Haida Gwaii‐Graham Island Lowlands (CO‐25; Yakoun [YAK], Datlamen [DAT], Tlell [TLE], Sangan [SAN]; Figures 4b and 5b) was highly supported (Figure S8b,c), which was not apparent with neutral markers.

In the Thompson region, genetic clusters also typically corresponded with CUs regardless of marker type used (Figures 4c,d and S7b; see Figure S9 for cluster support). One notable observation is a split between Louis [LOU], Lemieux [LEM], Fennell [FEN] and Barriere [BAR] in North Thompson (CO‐09) and the remaining five sampling locations in CO‐09 (Figure 5a), which can be seen with both neutral and outlier loci (Figure 4c,d). In South Thompson (CO‐08), Salmon River [SAL] was relatively dissimilar to other populations in the same CU, as evidenced by the longer branch length (Figures 4c,d and 5a), and Sinmax Creek [SIN] also diverged from these populations especially when GEA outliers were used (Figures 4d and 5b). Overall, the correlation between cophenetic distances among sampling locations across trees was significant (neutral vs. GEA: *r* = 0.36, *p* = 0.001; neutral vs. RDA: *r* = 0.31, *p* = 0.003).

**FIGURE 6:**
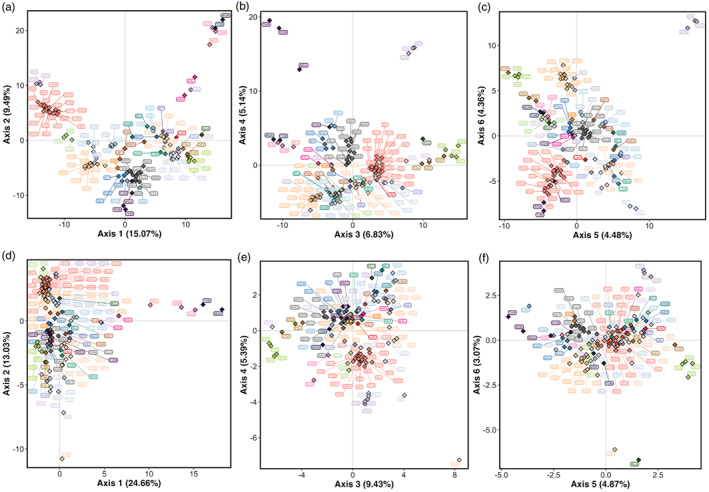
DAPC scatterplots for the BC region with (a–c) neutral and (d–f) GEA outlier SNPs. Diamonds represent the centroids of groups (see Figure S11 for plots with points around centroids) coloured according to the CU to which they are designated. CU, conservation units; DAPC, discriminant analysis of principal components; GEA, genotype–environment association; SNP, single‐nucleotide polymorphism.

**FIGURE 7:**
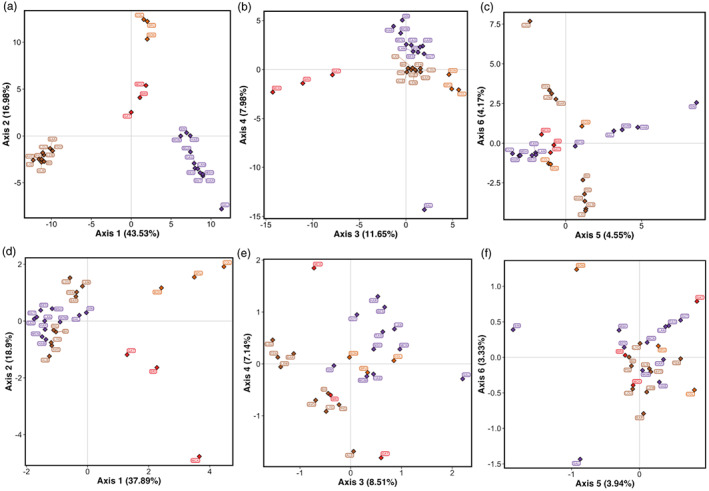
DAPC scatterplots for the Thompson region with (a–c) neutral and (d–f) GEA outlier SNPs. Diamonds represent the centroids of groups (see Figure S12 for plots with points around centroids) coloured according to the CU to which they are designated. CU, conservation units; DAPC, discriminant analysis of principal components; GEA, genotype–environment association; SNP, single‐nucleotide polymorphism.

Discriminant analysis of principal components using either neutral or outlier SNPs generally showed a high degree of similarity between populations within the same CU in both regions (Figures 6 and 7; Figure S10; see also https://axuereb.shinyapps.io/Coho_DAPC/) and corroborated results from the neighbour‐joining clustering. In BC, populations in the Middle Skeena CU (CO‐33; Morice River/Toboggan [MOR/TOB]) and Lower Skeena CU (CO‐34 Sustut/Damshilgwit [SUS/DAM]) strongly separated from the rest of the BC populations, as did populations in Lillooet (CO‐04; Birkenhead/Poole Creek/Upper Birkenhead [BIR/POO/UPB]) and Lower Fraser (CO‐47; e.g. Alouette/Chilliwack River/Railroad [CHW/SIL/RAI]). Additional axes revealed strong separation of West Vancouver Island populations (CO‐17; Robertson/Thornton/Maggie [ROB/THR/MGG]) from other BC populations (Figure 6b). DAPC results were also similar regardless of the marker type used (Figures 6 and S10a–c). Notably, in the Northern Coastal Streams (CO‐30), Canoona [CAN] displayed stronger separation from the other populations in the same CU with GEA and RDA outliers compared with neutral SNPs. In the Thompson group, the same patterns were observed as with neighbour‐joining clustering: (1) Salmon River [SAL] showed marked separation from other South Thompson (CO‐08) populations with both neutral and outlier SNPs, (2) Sinmax Creek [SIN] also separated from the remaining CO‐08 populations with outlier SNPs and (3) two groups were apparent in North Thompson (CO‐09) separating LOU/LEM/FEN/BAR from the remining CO‐09 populations with both neutral and outlier SNPs (Figure 7; Figure S10d–f).

## 4 DISCUSSION

The increasing feasibility of generating genomic data sets provides an opportunity to incorporate more precise estimates of population genetic statistics, inferences of population genetic structure across multiple spatial scales, and information about local adaptation into conservation and management planning across a range of species. The general approach used in this study is not limited to aquatic species and has been applied to delineate different types of CUs, including ESUs, MUs and/or AUs for other taxa including terrestrial mammals (Barbosa et al., 2018), plants (Silva et al., 2020) and amphibians (Forester et al., 2022) of conservation concern. In cases where CUs have already been established, the power of large SNP data sets can be harnessed to validate and potentially adjust existing boundaries to improve management approaches (Forester et al., 2022; Waples et al., 2020). Although we found a strong match between genomically derived units based on either neutral or outlier loci and currently defined CUs for Coho salmon in Canada, other studies have raised important questions concerning the possible revision of CUs for Chinook salmon (*O. tshawytscha*) and steelhead (*O. mykiss*) after strong genomic associations with adult migration timing that may not be reflected in the current configuration of CUs were identified (Waples et al., 2022; Waples & Lindley, 2018). Consequently, it would be advisable to take advantage of large SNP data sets, when available, to revisit CU definitions that were made in the absence of genomic information to ensure that management objectives are being met. While demonstrating local adaptation may remain challenging for many species, for example if adaptive traits are not controlled by loci of large effect or if genomic resources (e.g. annotated reference genomes) are lacking, the candidate‐based approach used here can be broadly applied to assess how spatial patterns of genetic structure and GEAs may or may not corroborate existing CU definitions and inform potential modifications to management or conservation strategies going forward.

### 4.1 Broad scale patterns of genetic structure

Our results from analyses of population genetic structure across the Canadian distribution of Coho salmon corroborated previous findings. Namely, our findings support the strong differentiation of the Thompson River (CO‐07, CO‐08 and CO‐09) and interior Fraser (CO‐48) populations from all other populations, including those in the lower Fraser River (CO‐47; Small, Beacham, et al., 1998), as well as IBD driven by northward expansion following Pleistocene glaciation (Rougemont et al., 2022). Distinction of populations from the Thompson River, which flows into the Upper Fraser, has also been reported in other species such as Chinook salmon (Beacham et al., 2003) and Sockeye salmon (Wood, 1995), and is hypothesized to be a result of postglacial colonization of the Upper and Lower Fraser drainages from different source populations (Small, Beacham, et al., 1998). Previous demographic modelling work also suggested that Thompson River populations evolved in isolation from the main distribution of Coho salmon in BC for a long period of time (Rougemont et al., 2020). Furthermore, these regions are separated by the Fraser River Canyon (CO‐5), which may serve as a barrier between spawning grounds for Thompson River and Lower Fraser populations (Wehrhahn & Powell, 1987), thus isolating populations on either side and supporting adaptive divergence between lower and interior (upper) regions as a result of differential selection pressures (Taylor & Mcphail, 1985). The Kawkawa Creek (KAW) population displayed a high degree of ancestry from both biogeographic regions. This population is located in the Fraser River canyon (CO‐5) and spawns in the Coquihalla River drainage, which flows directly into the Fraser River. The intermediate position of this population indicates a shared origin to the upper and lower Fraser river drainages.

The strong divergence observed between the Thompson river populations and all other populations in BC has implications for the definition of Coho salmon ESUs. While there are several definitions of ESUs in the literature (see Fraser & Bernatchez, 2001; Funk et al., 2012), all of them imply a substantial degree of biological distinctiveness and evolutionary independence. According to one of the most commonly used definitions, ESUs are defined based on substantial reproductive isolation among populations or groups of populations that represent ‘an important component in the evolutionary legacy’ (Waples, 1991), which includes both the evolutionary history and the evolutionary potential of a species (Waples, 1995). Barbosa et al. (2018) recommend the use of multiple markers (e.g. mitochondrial and nuclear markers) to define ESUs as distinct genetic units that diverged due to phylogeographic events. Here, we observed a distinct genetic split between the Thompson populations and all other populations, a pattern which is consistent with previous studies using SNP markers (Beacham et al., 2020), microsatellite and MHC markers (Beacham et al., 2001; Small, Withler, & Beacham, 1998), and mitochondrial DNA haplotypes (Smith et al., 2001). Furthermore, Rougemont et al. (2022) identified evidence for local adaptation to conditions related to long‐distance spawning migrations in the Thompson populations, with outlier SNPs associated with normalized distance displaying elevated allele frequencies in populations in the Thompson group compared with populations that undergo shorter migrations to spawning grounds. These outlier SNPs were found to be potentially associated with functions related to cardiac performance. Given the strong divergence of Thompson River populations from all other populations in BC resulting from historical colonization, contemporary isolation, and adaptation, our findings together with those of previous studies support the definition of at least two distinct ESUs in BC that represent an important component of genetic diversity for Coho salmon in Canada. These two genetic groups would also meet the criteria to recognize them as distinct DUs as defined by the Committee on the Status of Endangered Wildlife in Canada (COSEWIC; Green, 2005).

### 4.2 Comparison of genetic groups with current CUs (neutral markers)

The definition of CUs for Pacific salmon was an important first step in the implementation of the WSP in Canada (DFO, 2005). The intent of these units was to represent biologically meaningful entities of diversity for standardized monitoring of the status of all five wild salmon species and assessing impacts of actions taken to conserve and manage wild populations (DFO, 2009). Specifically, diversity was characterized according to a combination of ecological, life history and genetic components and subsequently partitioned into distinct units with defined geographic boundaries. For Coho salmon, genetic diversity was characterized using 10 microsatellite markers, leading to the definition of 44 CUs in Canada (Holtby & Ciruna, 2007; Wade et al., 2019). Given the potential for genome‐wide variant datasets with larger numbers of markers to detect more subtle genetic breaks, the aim of this current study was to validate the boundaries of the established CUs with a more powerful genomic dataset. Based on our data set comprising tens of thousands of neutral SNP markers, we detected genetic clusters that largely matched with the populations or groups of populations managed within each CU, globally lending support to the current geographic demarcation of Coho salmon CUs.

Despite overall concordance between genetic groups detected in this study and the previously defined CUs, we did observe genetic subdivisions within some of the established CUs as well as some populations that were more genetically similar to different CUs than to the one they were presently assigned to. In general, inconsistences between genetic groupings and CUs occur where CU boundaries do not adequately reflect underlying geographic discontinuities that may not have been apparent with a small set of genetic markers and a more constrained sampling effort. In one example, we observed a genetic break between populations sampled from Howe Sound‐Burrard Inlet (CO‐10): the three coastal populations in Burrard Inlet (Seymour, Capilano, and Chapman) formed a cluster that was distinct from the remaining four sampled populations in the same CU (Mamquam River, Tenderfoot Creek, Ashlu Creek, and Shovelnose Creek), all of which are tributaries to the Squamish River draining into Howe Sound. The Burrard Inlet populations were more genetically similar to populations in East Vancouver Island (CO‐13) across the Strait of Georgia than to the other populations in CO‐10. In the Nahwitti Lowland (CO‐15) in northern Vancouver Island, another genetic group consisting of the west coast Vancouver Island populations (Stephens Creek, Washlawlis Creek, Waukwaas Creek, and Marble River) and Quatsese River was clearly separated from populations in the same CU on the east coast of Vancouver Island across both methods. Marble Creek Coho may also be differentiated from other populations in this group. A similar pattern has also been observed for Chinook salmon, where individuals from Marble Creek were distinct from those in nearby locations (Beacham, Jonsen, et al., 2006).

In the central coast, there was some evidence to suggest potentially modifying CUs, but boundaries between genetic clusters were difficult to define precisely. Genetic clustering analysis revealed similarity among groups of populations managed across multiple CUs in the central coast of BC (e.g. CO‐30, CO‐27, CO‐29 and CO‐20). Previous work on Coho salmon population structure using microsatellites also failed to detect clear population structure in the central coast of BC (Beacham et al., 2011), and similar patterns have been observed in other salmonids, including Sockeye (Beacham, McIntosh, et al., 2006; Wood, 1995) and Chum salmon (Beacham et al., 2009). The complex landscape and glaciation history in this region likely contribute to the lack of marked geographic structuring among populations of Coho and related salmon species in the central coast region. For example, population structure for Sockeye and Chum salmon was consistent with colonization from multiple sources, including from refuge populations on Haida Gwaii (Warner et al., 1982). Moreover, it has been suggested that some Coho populations are derived at least partially from southern and island refugia (Small, Withler, & Beacham, 1998). Therefore, contemporary patterns of population structure for Coho and other related salmon species in the central coast of BC, in which some areas were previously unglaciated, may reflect postglacial colonization and secondary contact from a patchwork of local refugia.

Patterns of genetic structure in the Fraser River drainage basin showed that all sampled populations in the Lower Fraser (CO‐47) clustered strongly together. Within the Thompson region, we detected well‐supported genetic clusters separating the Lower, South and North Thompson, and interior Fraser populations corresponding to CUs (CO‐07, CO‐08, CO‐09 and CO‐48, respectively). One exception was Salmon River (SAL) in South Thompson (CO‐08), which was clearly separated from the remaining South Thompson populations with DAPC and formed a unique branch on the neighbour‐joining tree. This distinction of Coho from Salmon River has not been reported previously, suggesting that our data set was able to identify more subtle differentiation than was previously detectable. Although significant enhancements were made in Salmon River to mitigate precipitous declines in Coho populations in the Thompson River drainage, these efforts involved releasing hatchery‐reared juveniles that originated from the same location (Irvine et al., 1999) and are therefore not expected to lead to population divergence. However, reductions in population size and subsampling of the gene pool for enhancement may lead to a loss of genetic diversity and strong genetic drift in this population (Ryman & Laikre, 1991; Waples et al., 2016). Indeed, we detected very low genetic diversity (H_s_) in this population compared with other sampled populations in this CU and across all populations.

### 4.3 GEAs and outlier SNPs

Previous analyses uncovered important environmental drivers of local adaptation in Coho salmon populations across its entire North American distribution (Rougemont et al., 2022). Here, we used a similar approach based on GEAs to identify SNPs potentially under selection within the two aforementioned ESUs independently. These SNPs were subsequently used to define CUs according to patterns of putatively adaptive genetic variation (Funk et al., 2012). Although reduced representation methods such as GBS do not sample the complete genome and may inherently miss important adaptive loci (Lowry et al., 2017), we detected numerous outliers associated with migratory distance (here, normalized distance), temperature and precipitation, especially in the BC ESU. Indeed, migration distance was the most important variable identified in the recent range‐wide analysis of local adaptation in Coho (Rougemont et al., 2022) and its importance as a selective factor has been reported for other salmonid species that undergo spawning migrations (Hecht et al., 2015; Micheletti et al., 2018; Moore et al., 2017). Both temperature and precipitation have been identified as important environmental predictors of genetic variation for Arctic char (*Salvelinus alpinus*) in marine and freshwater habitats (Dallaire et al., 2021) and are likely to amplify stressors associated with long‐distance migrations for anadromous salmonid species (Flanagan et al., 2018; Gilbert & Tierney, 2018; Micheletti et al., 2018). While their specific impact is uncertain, the detection of strong correlations between candidate loci and environmental variables suggests that local adaptation is occurring at the geographic scale studied, and thus conservation planning should target the protection of adaptive genetic diversity in this region (Flanagan et al., 2018). Future studies using whole‐genome sequencing data will be valuable for improving our understanding of the genomic basis of local adaptation in Coho.

In the Thompson region, populations of Coho salmon are also known to have undergone significant population declines (Irvine & Bradford, 2000) leading to severe bottlenecks and strong genetic drift (Rougemont et al., 2020). Divergence due to drift in small populations could have led to spurious associations with spatially varying environmental conditions and could explain why so few outlier loci were discovered by GEAs in the Thompson, especially with higher cut‐off limits. Given these demographic trends, it may be difficult to disentangle whether differentiation at outlier loci is driven by selection or genetic drift.

A common approach in GEA studies is to consider the overlap of SNP outliers detected by multiple methods (e.g. RDA and LFMM), with the aim of reducing false positive detections and increasing confidence in the retained SNPs (Narum & Hess, 2011). However, the performance of different methods that aim at detecting targets of selection depend on the assumed scenarios of demography and natural selection. These often differ from one method to another and are likely oversimplifications of the true demographic scenarios, so that combining approaches may actually restrict detections to only those variants subjected to the strongest effects of selection (Forester et al., 2018). Consequently, we retained the full set of unique outlier SNPs detected by both RDA and LFMM, and also considered the outliers detected by RDA alone since simulation work has shown that retaining only RDA outliers (compared with LFMM) was the best approach to maximize true positive detections while maintaining relatively low false positive detections under different demographic scenarios (Forester et al., 2018). Overall, observed patterns were comparable using both approaches, suggesting that our results using all outlier SNPs were not affected by potential false positives.

### 4.4 Defining conservation units with putatively adaptive markers

The wild salmon CUs in current use were defined based on ecological and (neutral) genetic distinctiveness as indirect measures of reproductive isolation that serve as proxies for local adaptation (DFO, 2009). Using SNP‐environment associations with large genomic data sets allows for the incorporation of putatively adaptive information into CU definitions more directly, which is an important goal for conservation planning (Flanagan et al., 2018; Funk et al., 2012, 2019). Indeed, several studies have shown considerable differences in patterns of population genetic structure when using different marker types, including in fishes such as Atlantic cod (*Gadus morhua*; Bradbury et al., 2013; Hemmer‐Hansen et al., 2013), Pacific lamprey (*Entosphenus tridentatus*; Hess et al., 2013), European lamprey ecotypes (*Lampetra fluviatilis* and *L. planeri*; Rougemont et al., 2017) and Atlantic herring (*Clupea harengus*; André et al., 2011). While genome‐wide genomic variation may be a suitable predictor of local adaptation in many cases (Fernandez‐Fournier et al., 2021; Kardos et al., 2021), distinguishing between neutral and adaptive genetic markers and integrating attributes of both, when possible, could assist in implementing solutions that aim at preserving a range of evolutionary processes (Hanson et al., 2020; Xuereb et al., 2021).

Overall, our analyses of population genetic structure based on outlier loci generally revealed concordant results to those based on neutral loci. These findings are analogous to previous results reported in anadromous Atlantic salmon (Moore et al., 2014), which share similar biological and life history characteristics with Coho that could explain the lack of differences observed between marker types. Namely, both species exhibit strong fidelity to spawning sites (Beacham et al., 2020; Hendry et al., 2004), thereby limiting gene flow and resulting in relatively isolated breeding populations. Straying (i.e. dispersal to non‐natal sites) that leads to gene flow may be constrained to nearby rivers that share similar environmental conditions, both because these rivers are likely to share the same chemical cues that are recognized by returning salmon (Dittman et al., 1996; Perrier et al., 2011) and because local adaptation may result in selection against migrants from rivers with different environmental characteristics (Nosil et al., 2005). As a result, molecular signatures of adaptation to local environmental conditions would be expected to closely match those of neutral divergence (Moore et al., 2014; Thibert‐Plante & Hendry, 2010).

The genetic architecture underlying adaptation may also explain the similarity of signals observed between different approaches. When adaptive traits are driven by one or a few loci or genes of strong effect, discrete groups associated with a given phenotype may be easily distinguishable. This was the case in steelhead (*O. mykiss*) and Chinook salmon (*O. tshawytscha*) populations, for which overall genetic differentiation based on genome‐wide SNP data largely reflected geography, but a single locus (*GREB1L*) strongly associated with migration timing distinguished populations characterized by premature and mature migration phenotypes, and called into question the need for CUs aimed specifically at conserving this important component of adaptive diversity (Prince et al., 2017; Waples et al., 2022; Waples & Lindley, 2018). On the contrary, we detected putatively adaptive SNPs based on subtle allele frequency differences associated with multiple environmental variables, such as migration distance, temperature and precipitation, which are likely to involve many physiological responses and complex traits characterized by a polygenic architecture. The polygenic nature of adaptation in Coho salmon was described in Rougemont et al. (2022), in which none of the detected outlier SNPs could be mapped onto genomic regions of strong effect reported in other salmonids. In this case, causal SNPs underlying polygenic traits will individually generate relatively weak signals that are not expected to be different from neutral population structure (Pritchard et al., 2010).

### 4.5 Recommendations and conclusions

Using one of the largest landscape genomics data sets for a nonmodel species, we investigated spatial patterns of genetic structure for Coho salmon populations in Canada to evaluate the delineation of CUs. Since CUs for managing Coho salmon populations have already been defined in their Canadian range using a combination of genetic (microsatellite and mtDNA), phenotypic, and ecological information, our aim was not to define a new set of CUs for this region. Instead, we aimed at validating and providing recommendations for potentially refining existing CU boundaries using a larger and more powerful genomic data set compared with that which was previously available. Following the framework proposed by Funk et al. (2012), we first assessed population genetic structure using the full filtered SNP data set, and subsequently identified candidate SNPs under selection to characterize units based on putatively neutral (i.e. MUs) and then adaptive (i.e. AUs) variation independently. At the broadest scale, our results supported the definition of two distinct ESUs (also two distinct DUs) for Coho salmon in BC, distinguishing the Thompson River (including Lower Fraser) populations from all other populations in BC. In the Thompson ESU, patterns of neutral population structure corresponded with the four existing CUs (CO‐07, CO‐08, CO‐09, CO‐48). The North Thompson CU (CO‐09) could be considered as two MUs, as we observed two discrete clusters consisting in one case of the southernmost populations (LOU/LEM/FEN/BAR) and in the other of the populations located further north (PIG/BII/LYO/AVO/ALB). The same patterns were observed with neutral and outlier SNPs, indicating a concordance between MUs and AUs within this ESU, except for Sinmax Creek [SIN] in North Thompson (CO‐08), which showed strong differentiation with outlier SNPs indicating potential consideration for a separate AU for this population.

In the BC ESU, we sampled from Coho populations that are currently managed in 31 of the 40 existing CUs. Overall, our results largely reflected CU definitions, suggesting no major restructuring. However, there are three areas in which additional MUs may be warranted based on evidence of substructure within the current CU boundaries. We propose: (1) separating Howe Sound and Burrard Inlet (populations currently managed together in CO‐10), (2) separating the west and east coasts of Northern Vancouver Island (populations currently managed together in CO‐15) and (3) separating the south‐west coast of Vancouver Island including Sooke [SOO] from the remaining East Vancouver Island‐Georgia Strait populations (CO‐13). Moreover, we suggest that boundaries may be refined between CO‐30 (North Coastal Streams), CO‐27 (Hecate Strait) and CO‐29 (Douglas Channel‐Kitimat Arm), whereby coastal populations in CO‐30 and populations in CO‐27 may be combined in a single MU, and the remaining CO‐30 populations could be combined with the CO‐29 populations to create another MU. Observed differentiation of Martin River [MTI] in CO‐30 also suggests that it may belong to a distinct MU, but more sampling in the lower portion of CO‐30 and in CO‐26 (Mussel‐Kynoch; unsampled in this study) would help to resolve this boundary.

Despite the generally similar patterns of population genetic structure using either neutral or outlier loci, we did observe a few inconsistencies that may warrant the definition of distinct AUs in BC. In the Johnstone Strait/Southern Fjords CU (CO‐12), all three sampled populations were differentiated, potentially indicating a need for distinct AUs. However, support for their distinction is unclear and sampling of other populations within CO‐12 would help elucidate genetic groups. We also suggest that while populations on Haida Gwaii clustered within three distinct MUs that match the current CU definitions (CO‐23, CO‐24 and CO‐25), populations in Haida Gwaii East (CO‐23) may warrant distinction as two AUs: the first one comprising Pallant [PAL] and Deena [DEE] and the second one comprising Copper [COP] on the east coast of Haida Gwaii along with Northern Haida Gwaii‐Graham Islands Lowlands populations (CO‐25).

Overall, our findings suggest that the current definition of CUs as genetically distinct entities was generally accurate despite the relatively limited genetic data available. In addition to genetic differentiation based on microsatellite markers, ecological, morphological and life history characteristics were also incorporated into the approach used to define CUs and the overall concordance observed with groups detected using putatively adaptive genetic variation suggests that they largely reflected local adaptation. The widespread agreement between patterns observed with either neutral or outlier SNPs also suggests that natural selection is an important driver of population structure for Coho salmon, where gene flow is restricted among populations that differ in environmental conditions. However, identifying those few cases that support the definition of AUs that are different from MUs is critical for making appropriate management decisions, such as ensuring that adaptively divergent populations are not used to supplement declining populations. Moreover, while CUs represented biologically meaningful groups with a high degree of differentiation among them, pronounced differentiation was also generally observed among populations within CUs. Strong site fidelity and homing behaviours exhibited by Coho and other salmonids (Quinn, 2018) is likely to restrict gene flow even between adjacent populations. Given this marked signal of population structure both at a broad scale and within CUs and the observed differences in patterns detected using neutral and outlier SNPs at a local scale, conservation and management decision‐making should consider differentiation at multiple levels. As previously argued by Funk et al. (2012), this includes taking into account differentiation at the highest level of ESUs, at the level of MUs and AUs within ESUs, as well as at the level of local populations themselves. In this study, using a more powerful genomic data set than was previously available, we validated the majority of currently used CUs and gained an improved understanding of the hierarchical levels of biological structure in this system. This new information should aid policymakers in their goal of ensuring the long‐term conservation of Coho salmon in Canada.

## CONFLICT OF INTEREST

We declare no conflict of interest.

## Supporting information


Appendix S1

## Data Availability

Raw data used in this project are deposited on NCBI under project PRJNA647050. Filtered VCF files are available at the Dryad Digital Repository: https://doi.org/10.5061/dryad.r4xgxd2gx. All scripts, environmental data and additional files for reproducing analyses may be accessed on GitHub at: https://github.com/amandaxuereb/CohoSalmonCUs.
