# Supplementary material for: Re‐evaluating Coho salmon (Oncorhynchus kisutch) conservation units in Canada using genomic data
Source: Evol Appl. 2022 Oct 18;15(11):1925–44. doi: 10.1111/eva.13489 (PMC9679250; doi:10.1111/eva.13489)
Supplement: Supplementary file 1 — Appendix S1 [file EVA-15-1925-s001.zip › eva13489-sup-0002-FiguresS1-S13.docx]

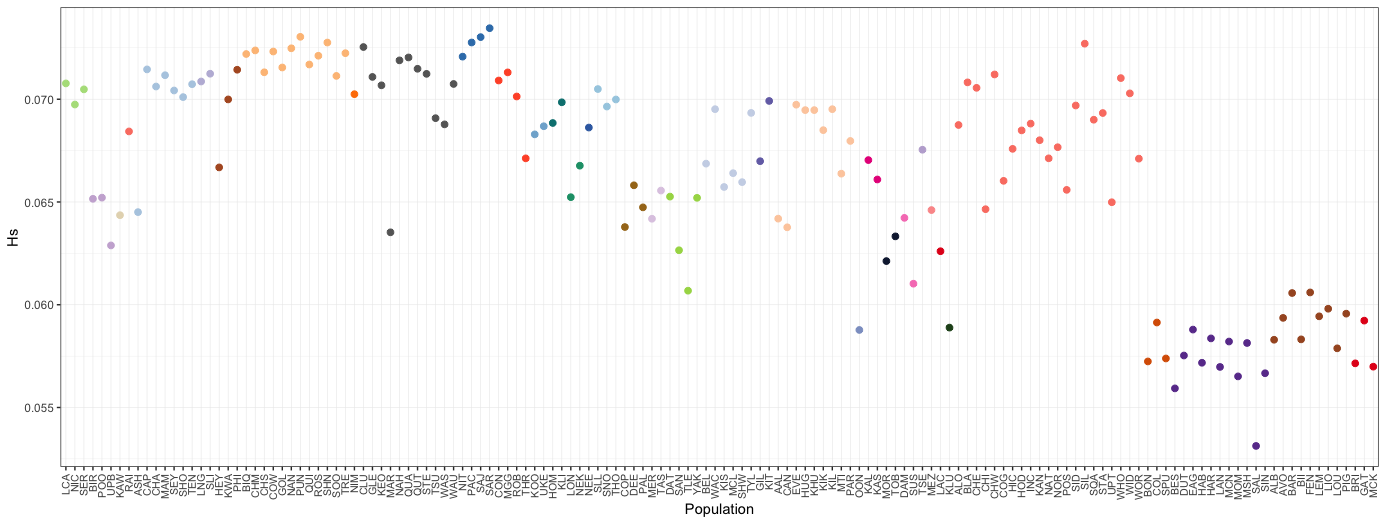


*BC*

*Thompson*

**Suppl. Fig. 1.** Mean expected heterozygosity (Hs) in all sampled populations. Points are coloured according to the CU in which populations are currently managed (see Fig. 1).


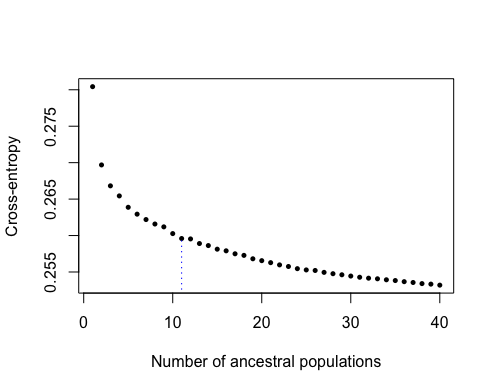
(A)

(B)


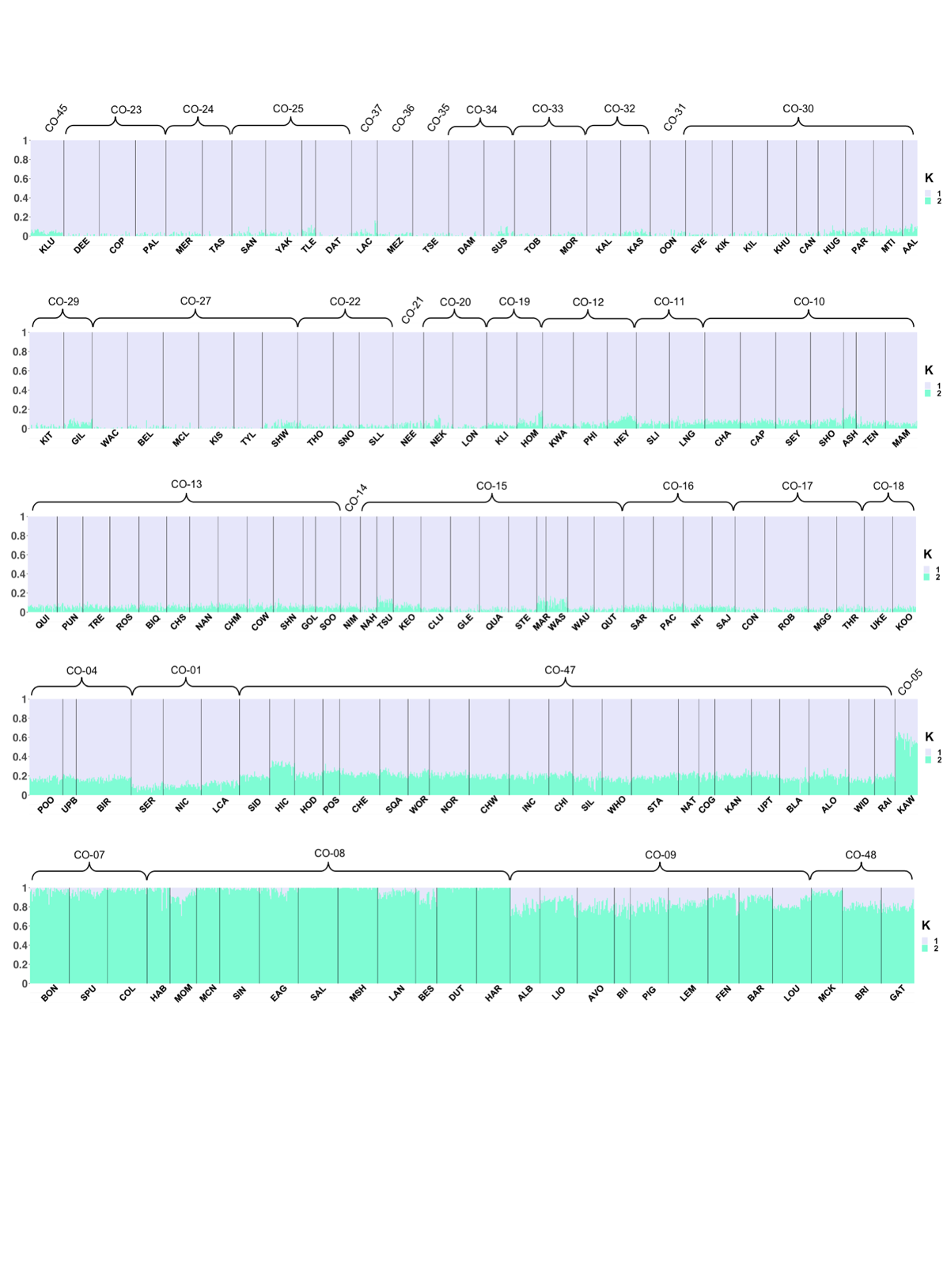


BC

Thompson

**Suppl. Fig. 2.** (A) Cross-entropy criterion for selecting optimal *K* (dotted blue line); (B)Admixture coefficients for all individuals grouped by sampling location (x-axis) in *K* = 2 genetic clusters. The current conservation unit designation for each sampling location is indicated by the curly brackets above the plots.


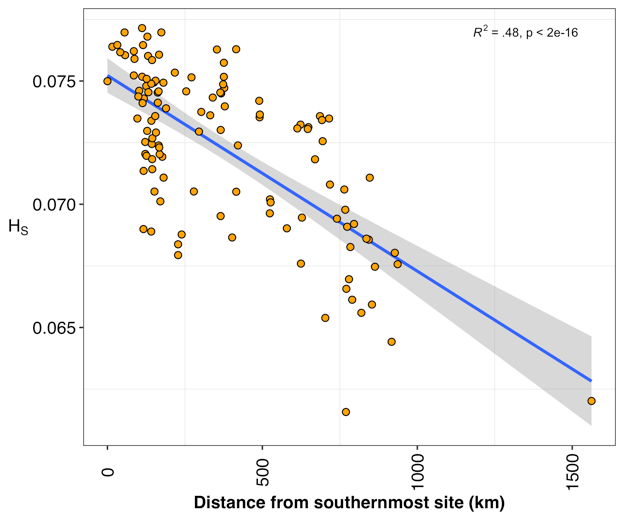

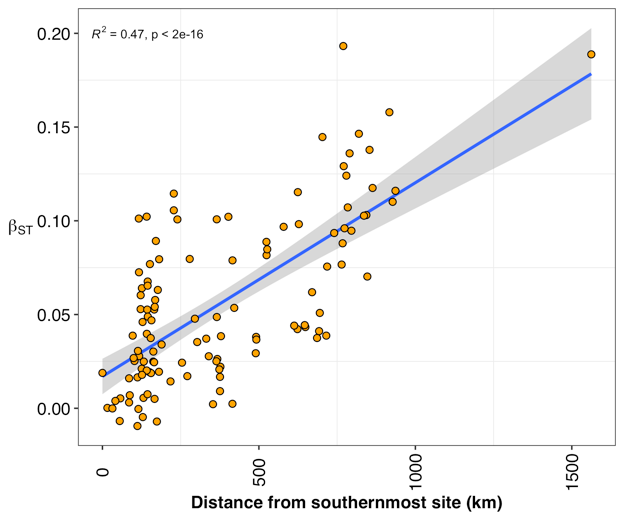


(A) (B)


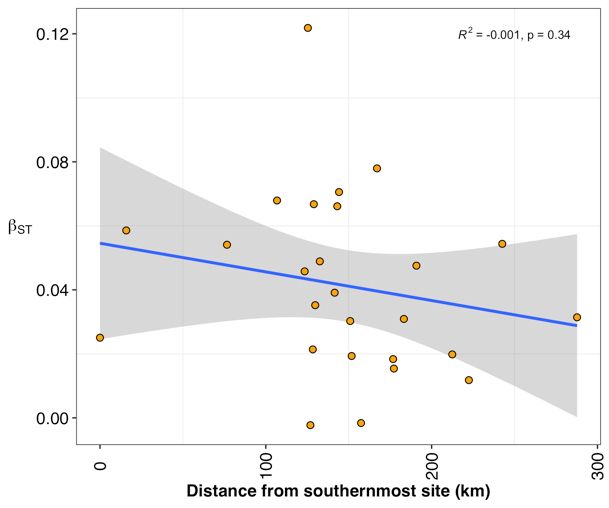

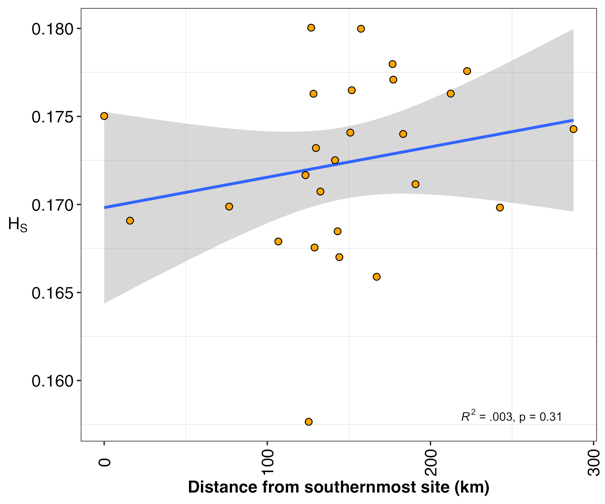
(C) (D)

**Suppl. Fig. 3.** Relationship between genetic diversity (Hs) (A, C) and genetic differentiation (BST) (B, D) and distance from the southernmost site in BC (top) and Thompson (bottom). The grey shaded region shows the 95% confidence intervals.

(A)


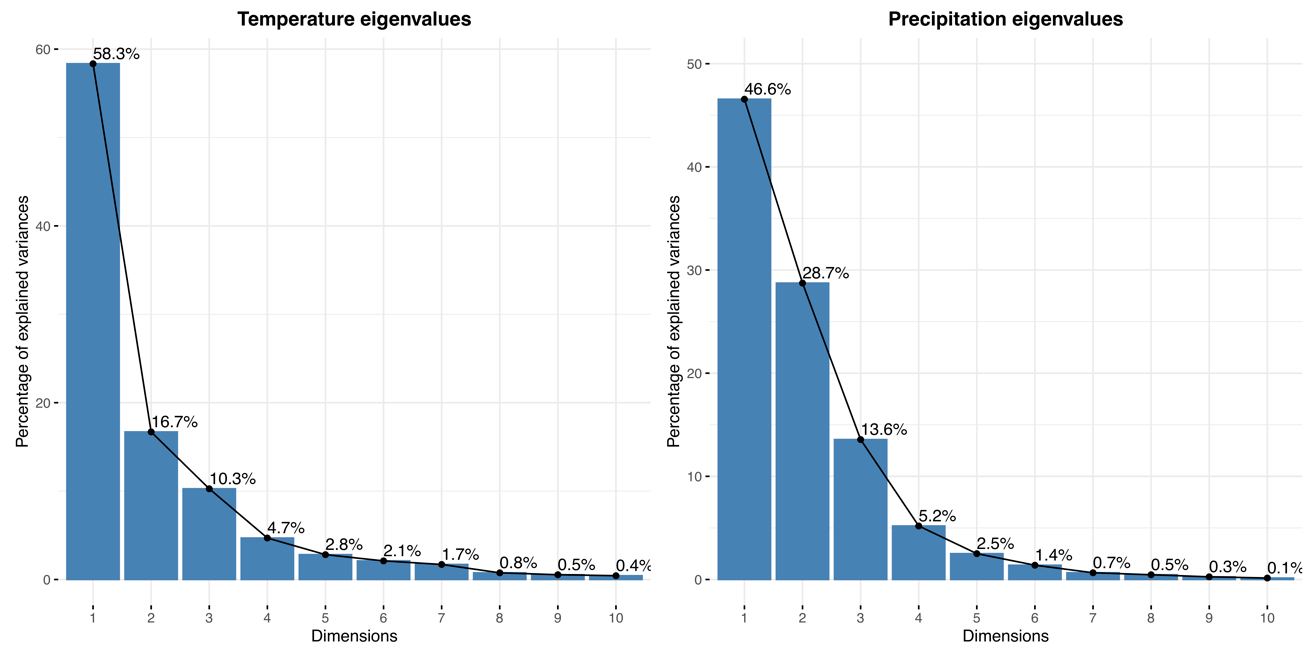


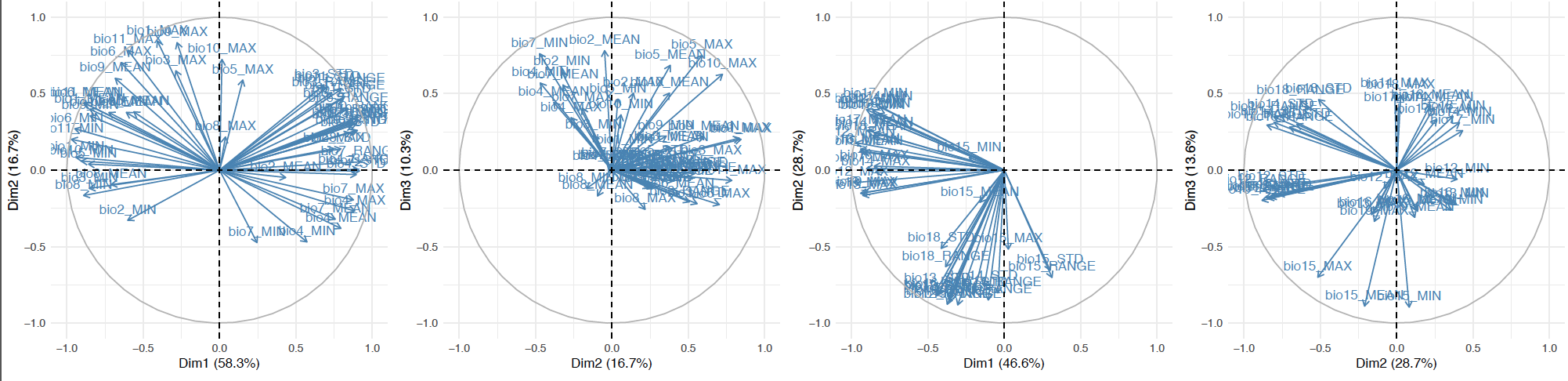
(B)


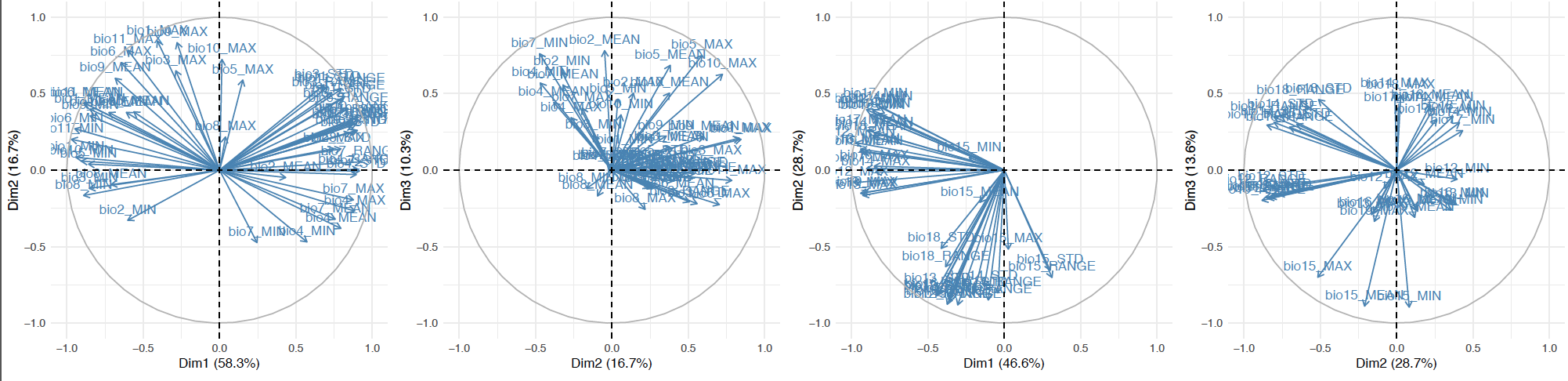


(C)

**Suppl. Fig. 4.** Proportion of variance explained by all eigenvalues from a principal components analysis on temperature and precipitation variables (A) and the loadings of all temperature (B) and precipitation (C) variables on the retained axes in the BC region.


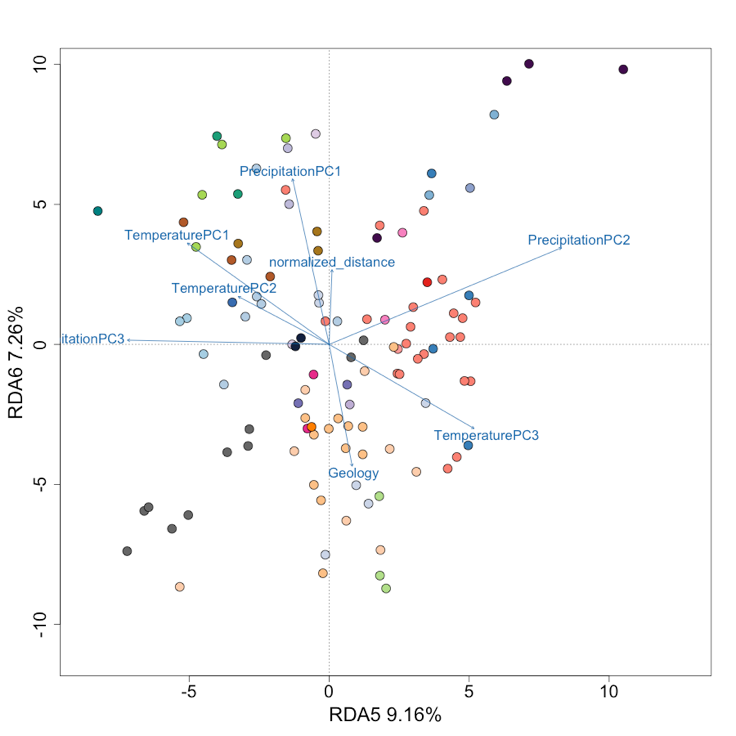

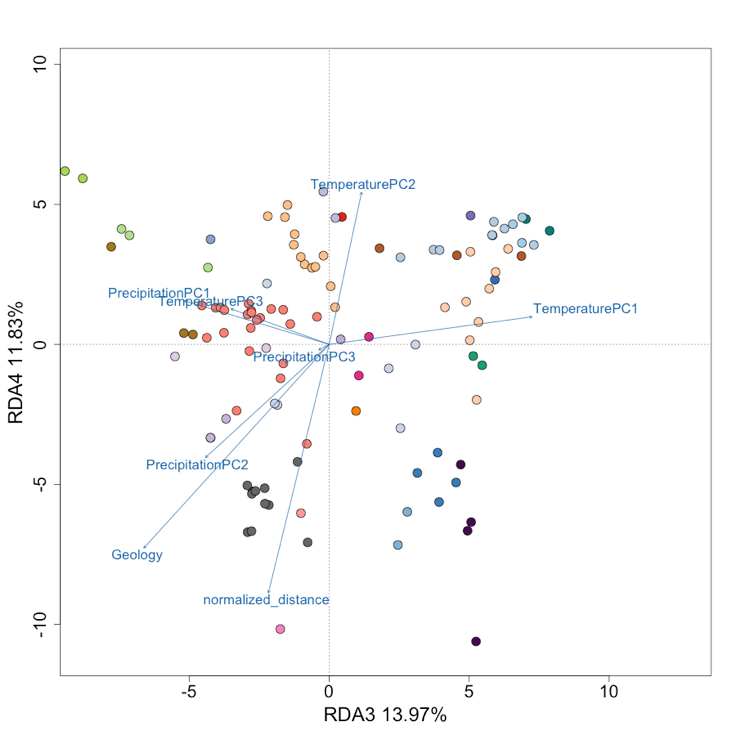


**Suppl. Fig. 5.** RDA results showing site loadings on axes 3 to 6 (points represent sampling locations coloured according to the CU in which they are currently managed, blue arrows indicate the environmental variables).


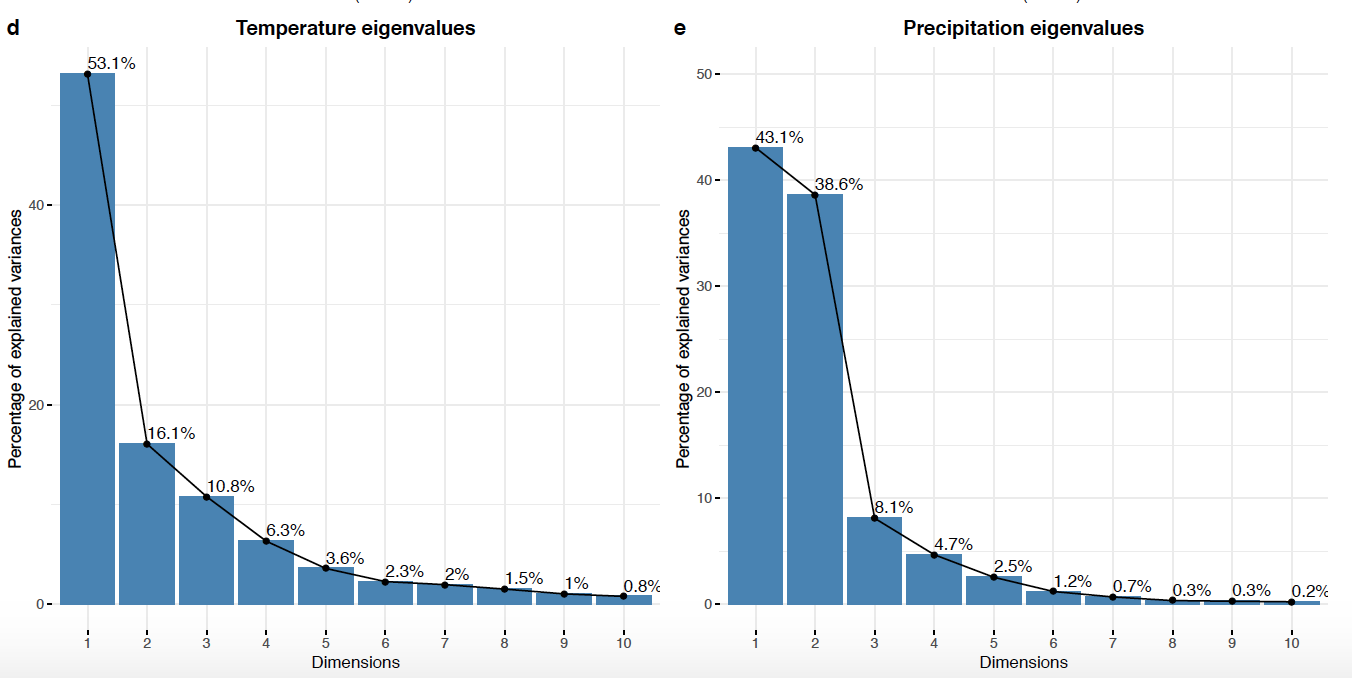
(A)

**
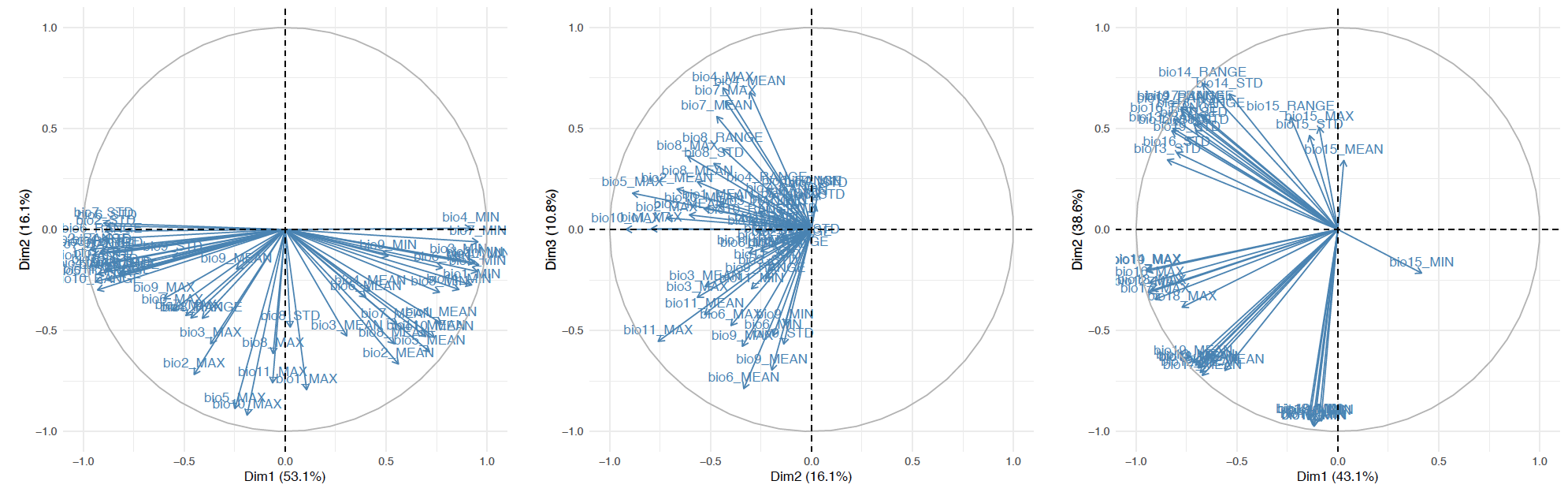
**

(B)

**
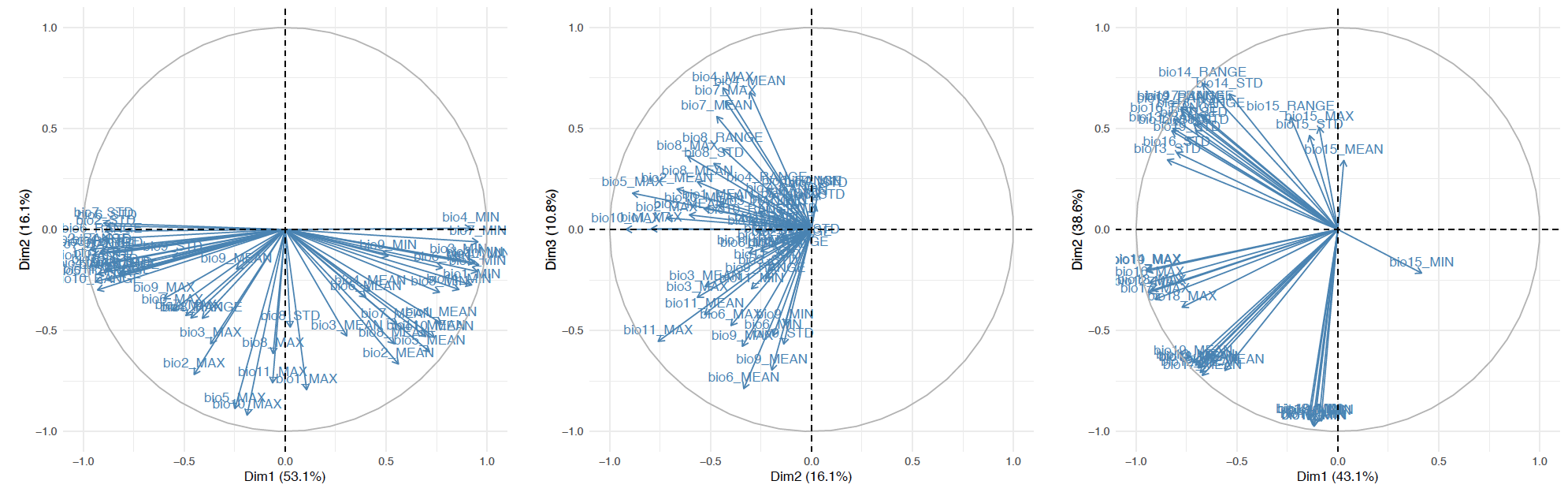
**

(C)

**Suppl. Fig. 6.** Proportion of variance explained by all eigenvalues from a principal components analysis on temperature and precipitation variables (A) and the loadings of all temperature (B) and precipitation (C) variables on the retained axes in the Thompson region.

**
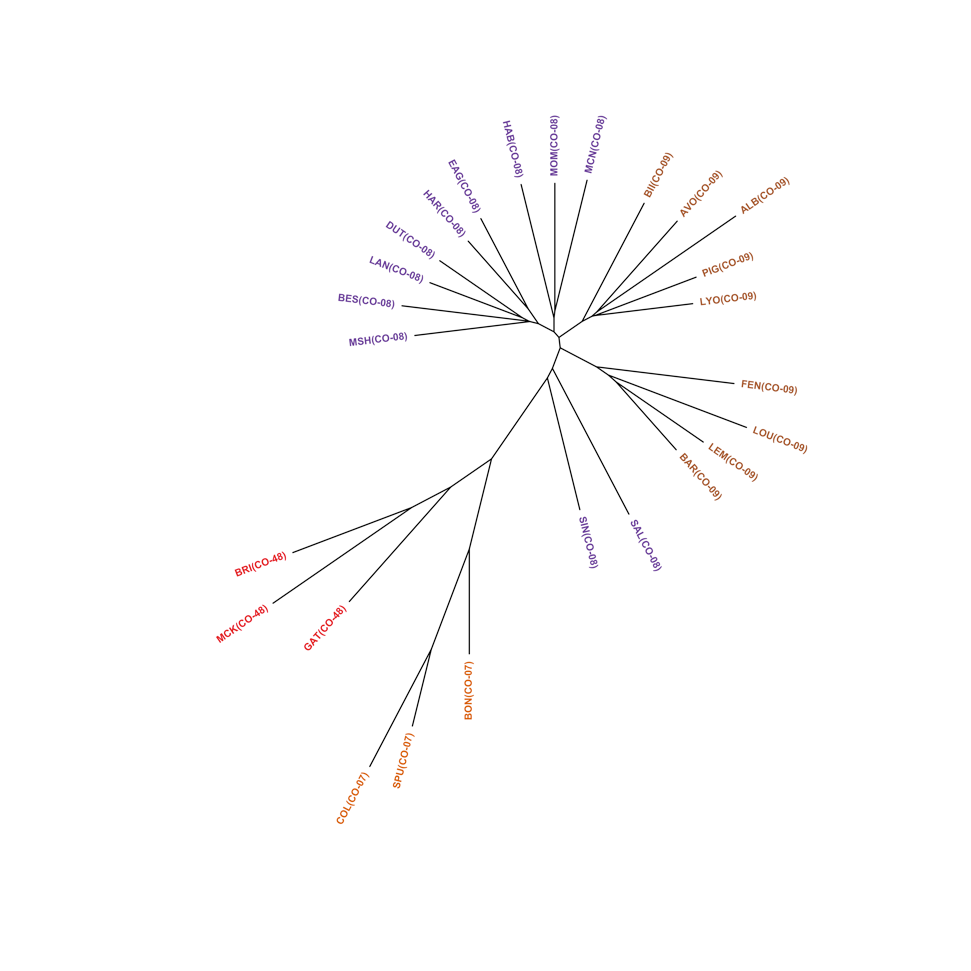
**

**
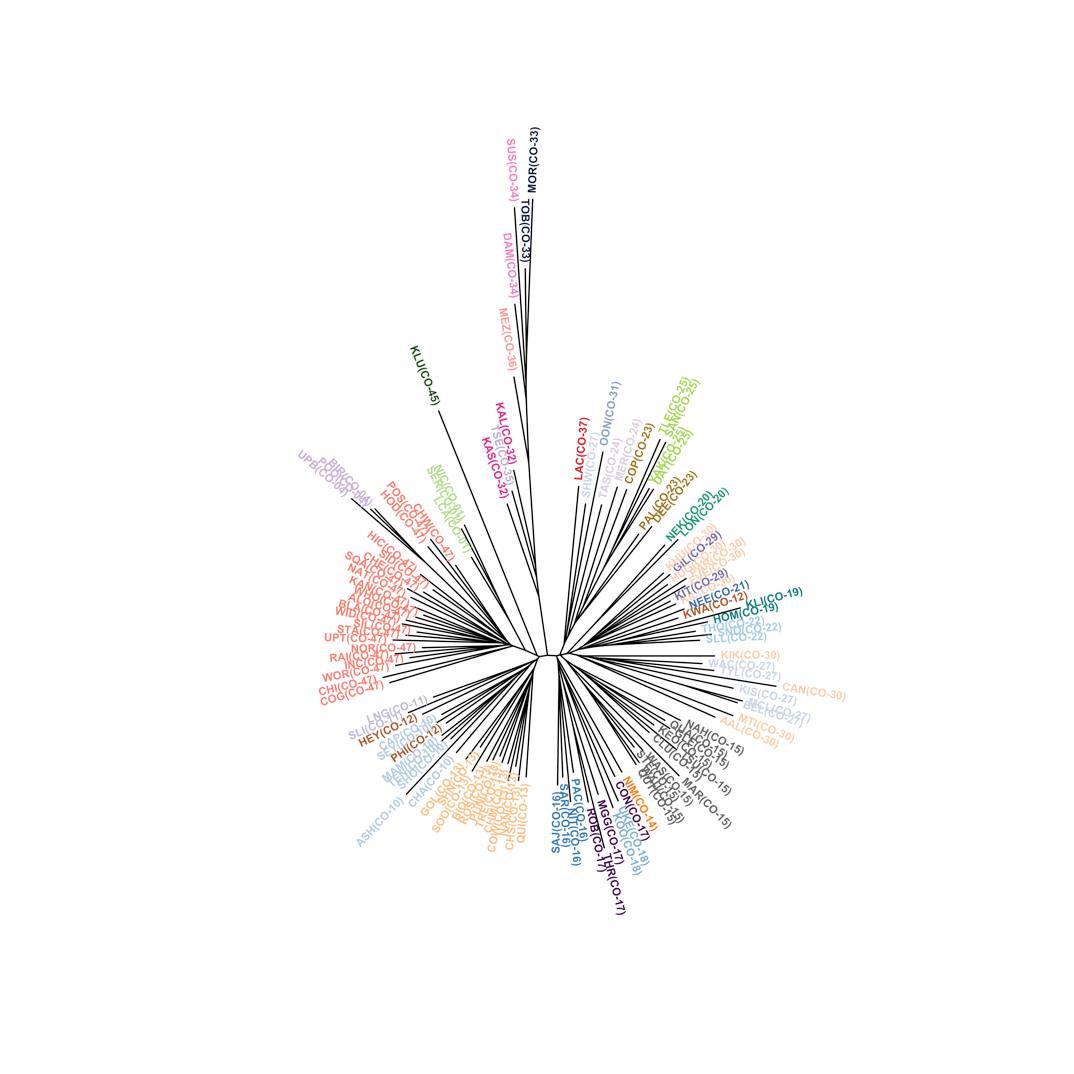
**(A) (B)

**Suppl. Fig. 7.** Unrooted neighbour-joining trees based on Cavalli-Sforza & Edward’s chord distance using candidate SNPs identified with RDA in (A) BC and (B) Thompson. Tips correspond to sampling locations and are coloured according to the CU they currently belong to.


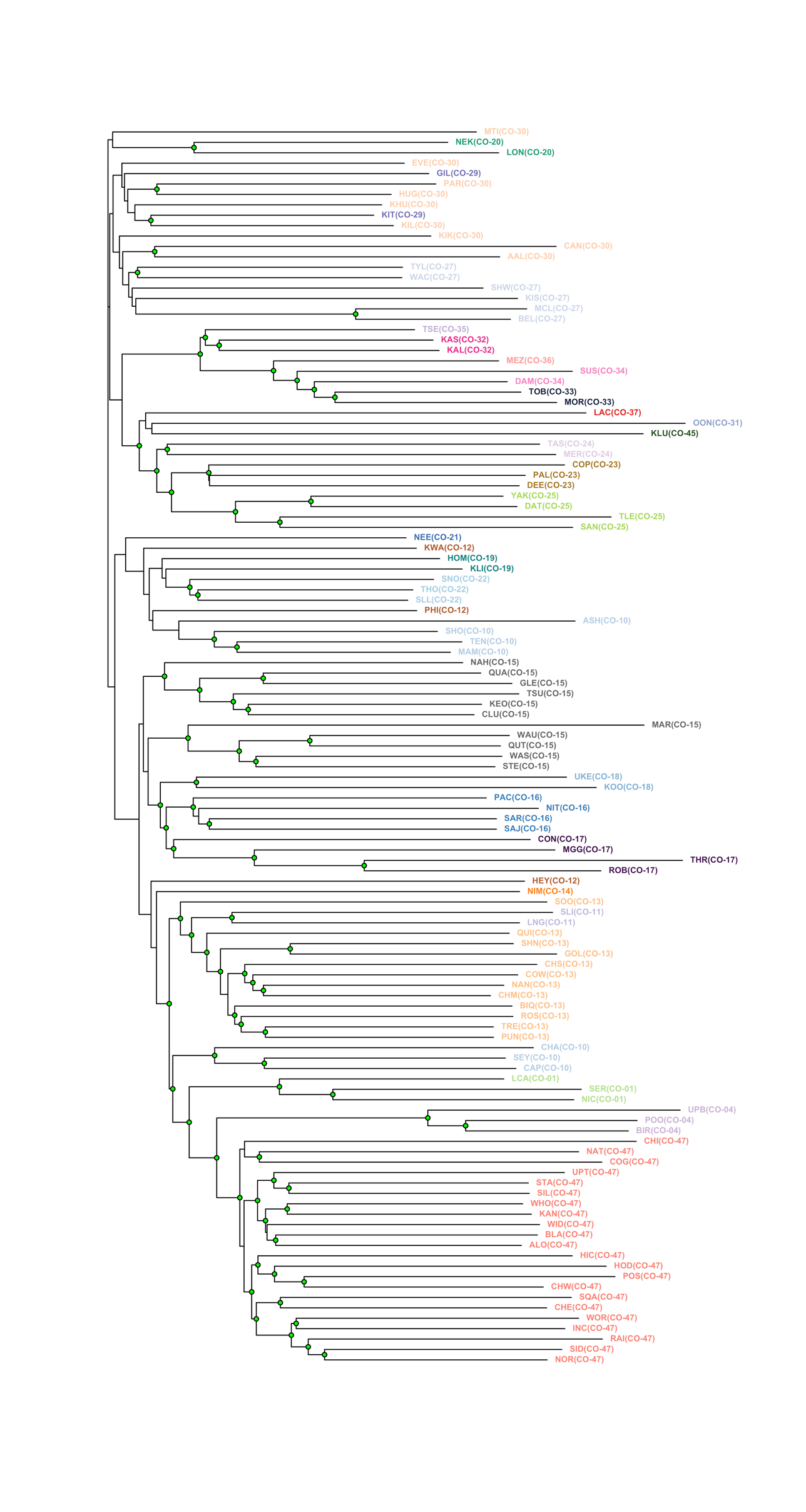
(A)


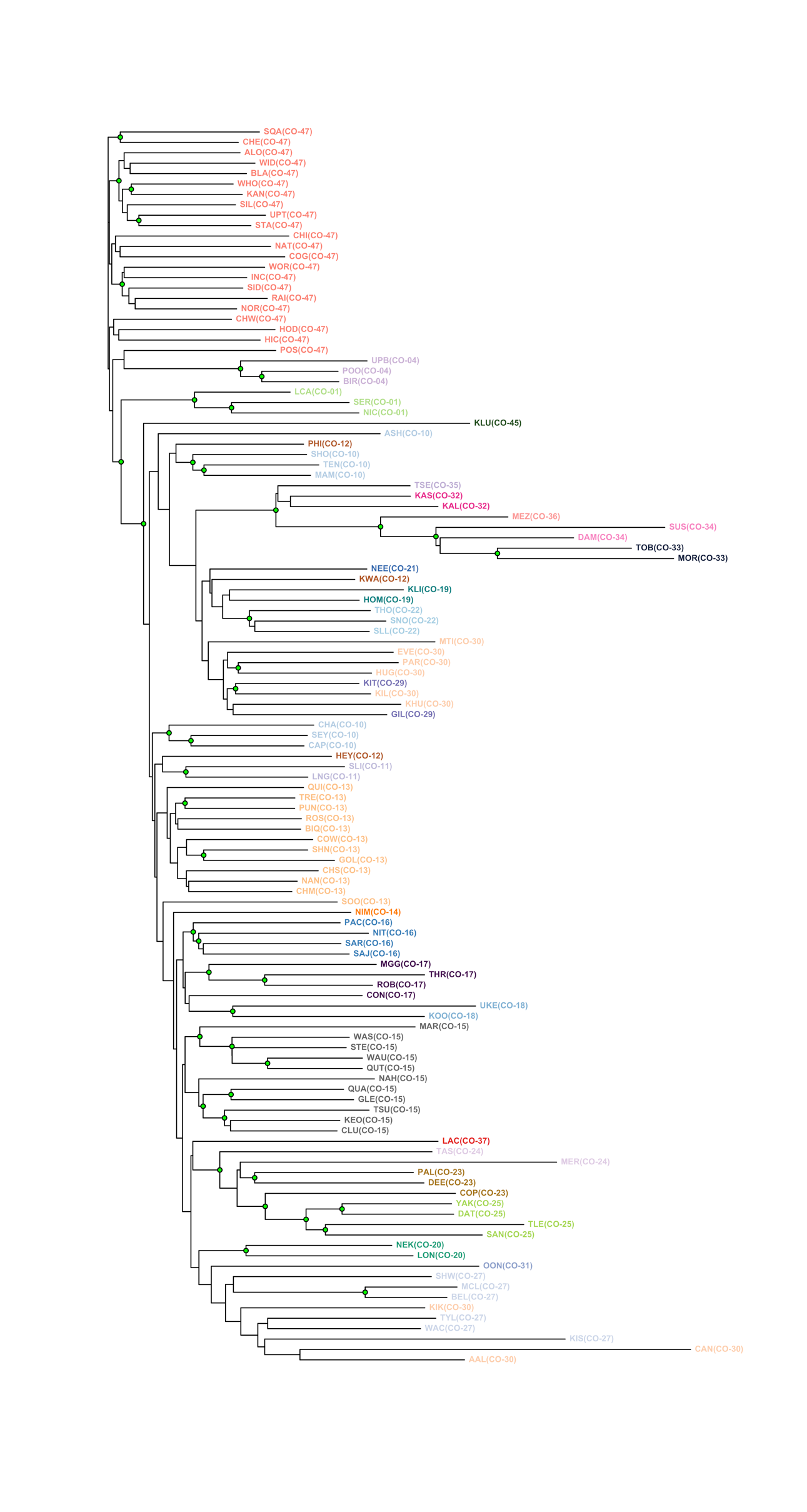
(B)


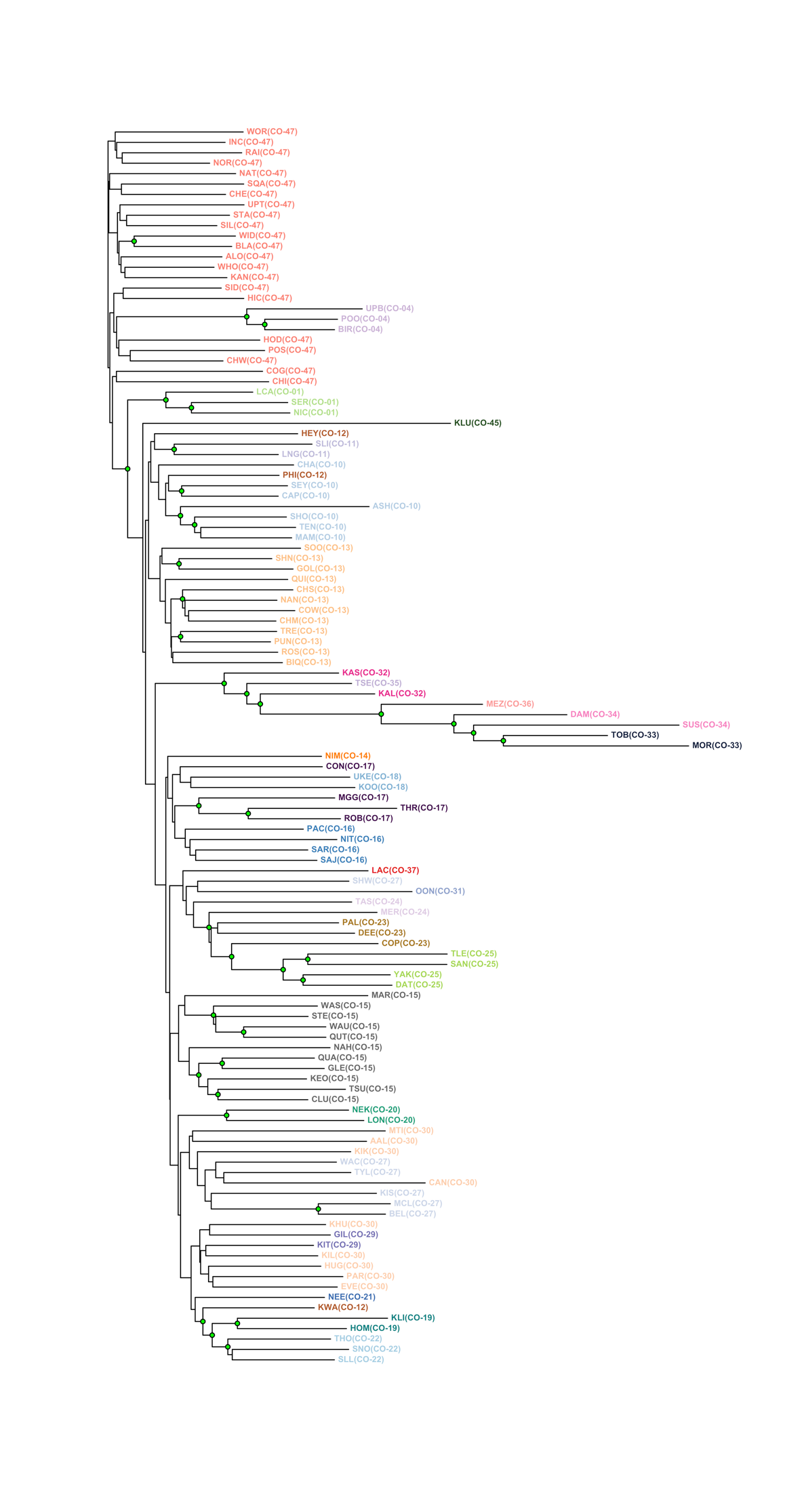
(C)

**Suppl. Fig. 8.** Unrooted neighbour-joining trees based on Cavalli-Sforza & Edward’s chord distance for the BC region using (A) neutral SNPs, (B) GEA outliers, and (C) RDA outliers. Green dots at nodes indicate cluster support >80%.


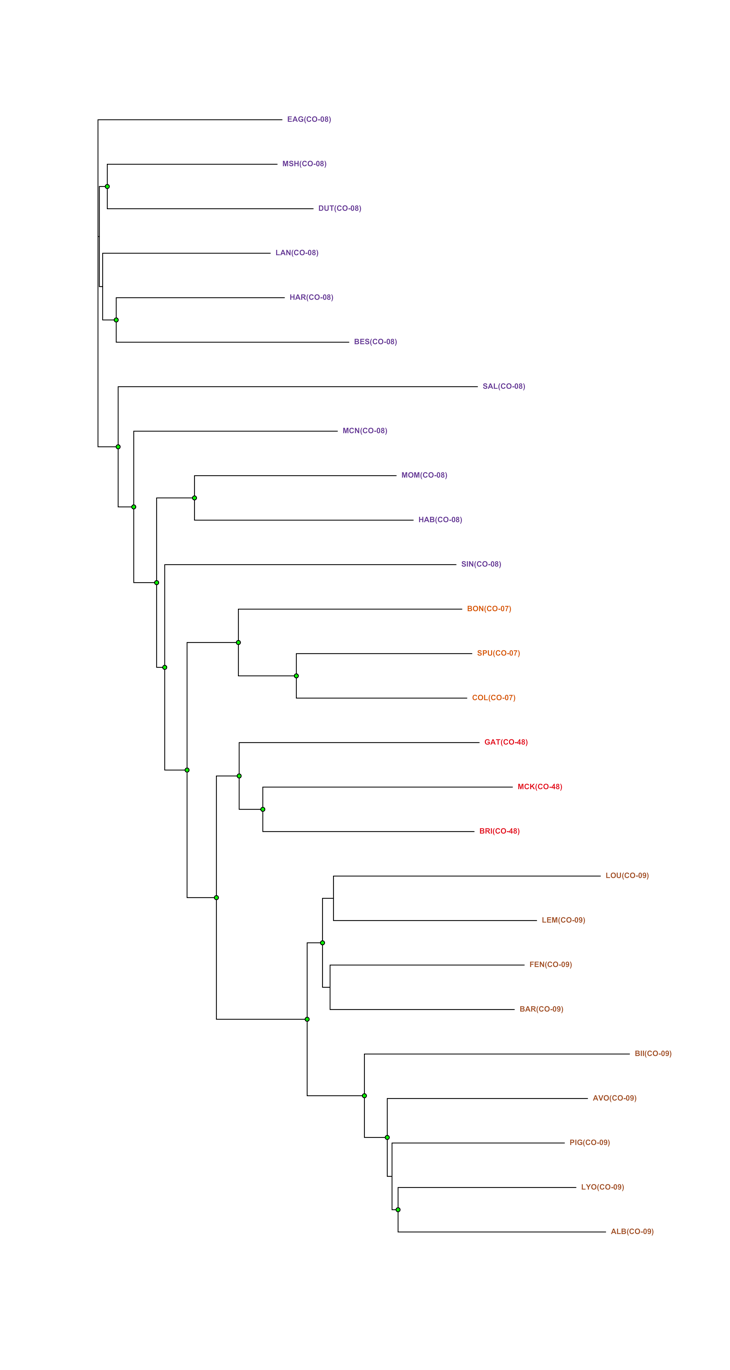

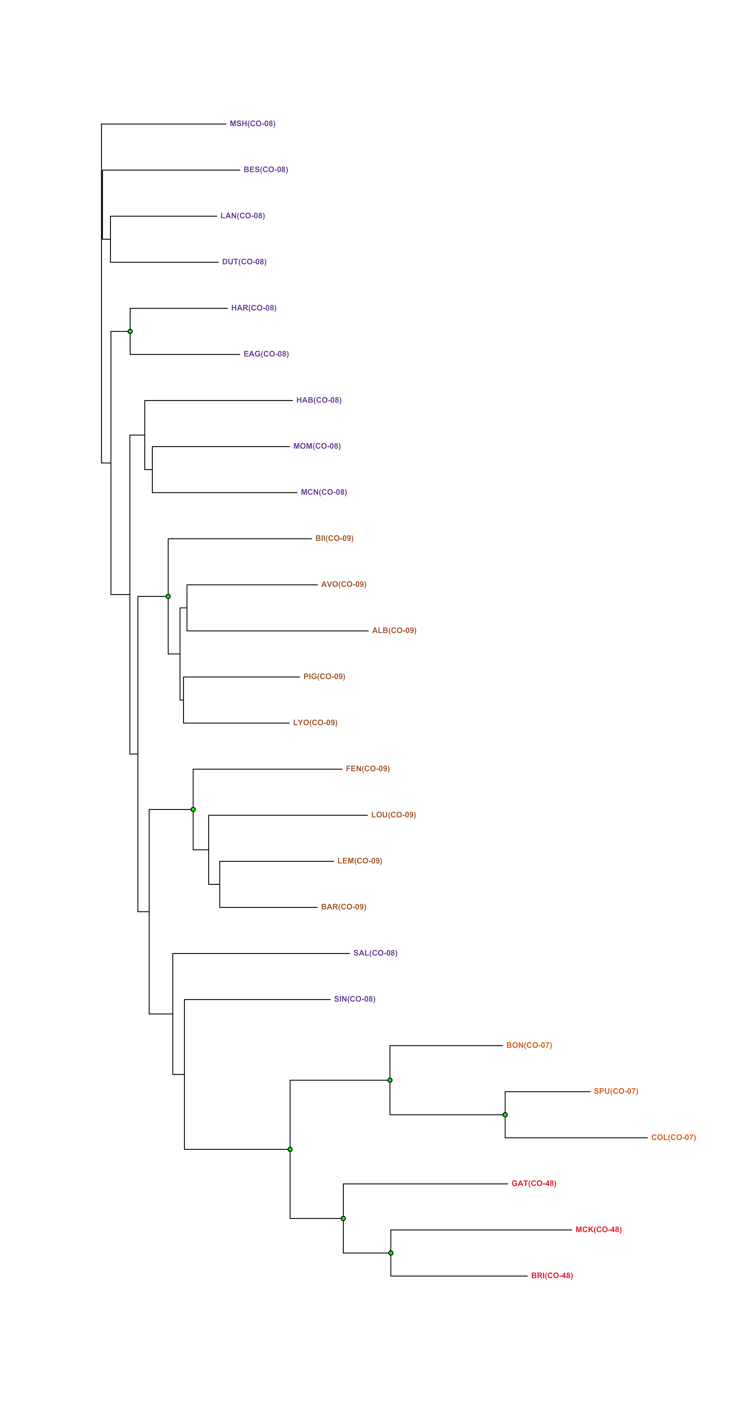

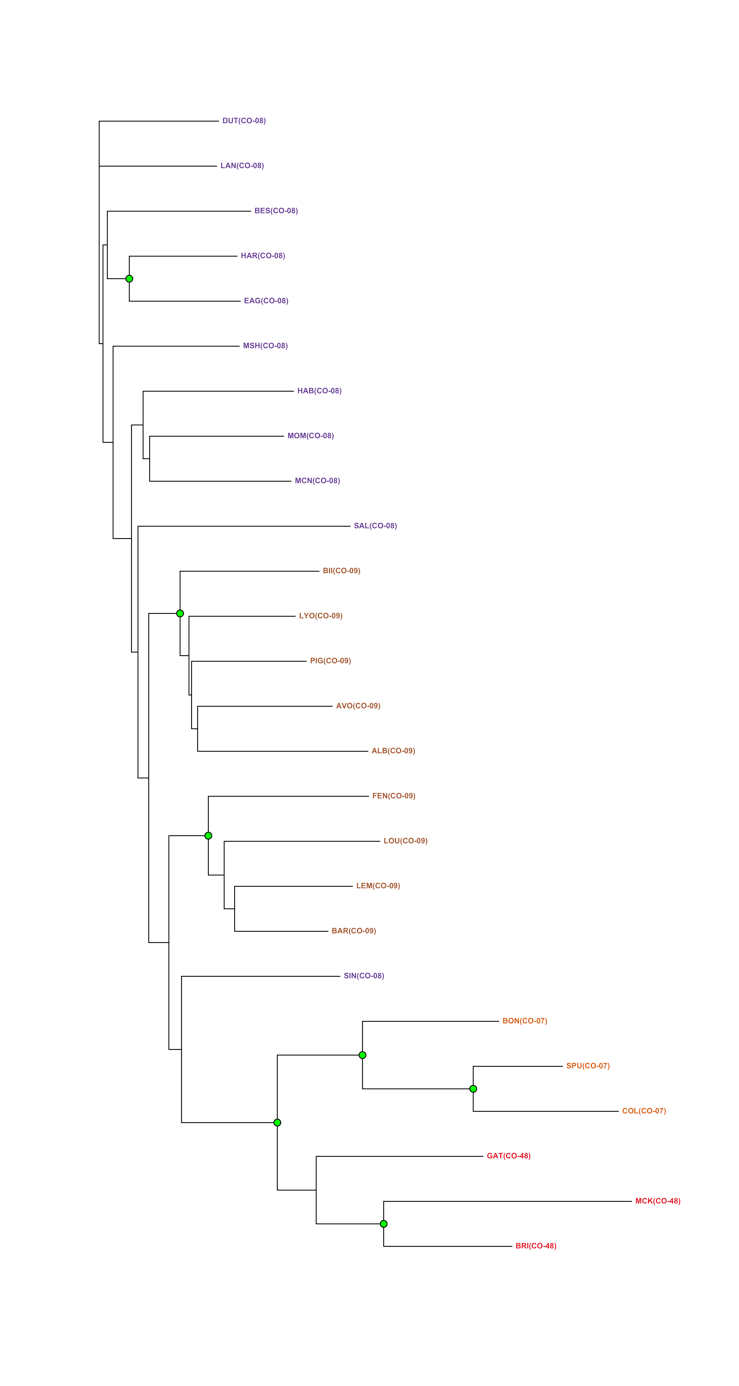


(A) (B) (C)

**Suppl. Fig. 9.** Unrooted neighbour-joining trees based on Cavalli-Sforza & Edward’s chord distance for the Thompson region using (A) neutral SNPs, (B) GEA outliers, and (C) RDA outliers. Green dots at nodes indicate cluster support >80%.


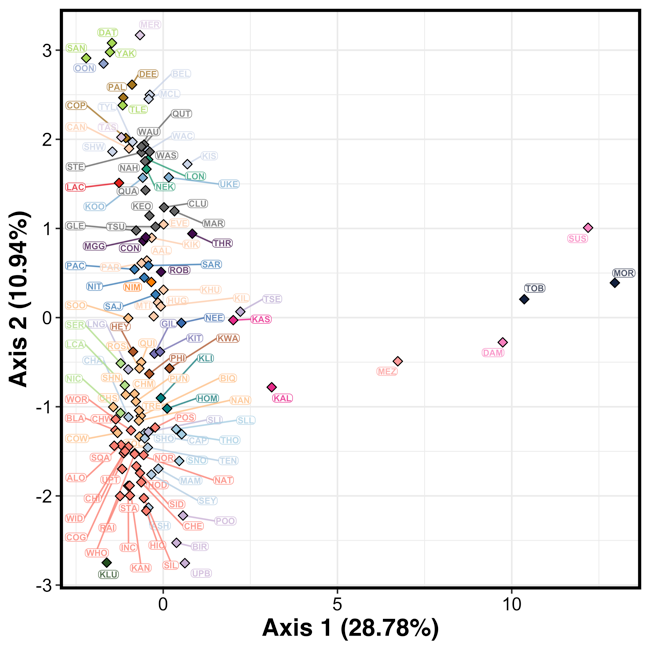

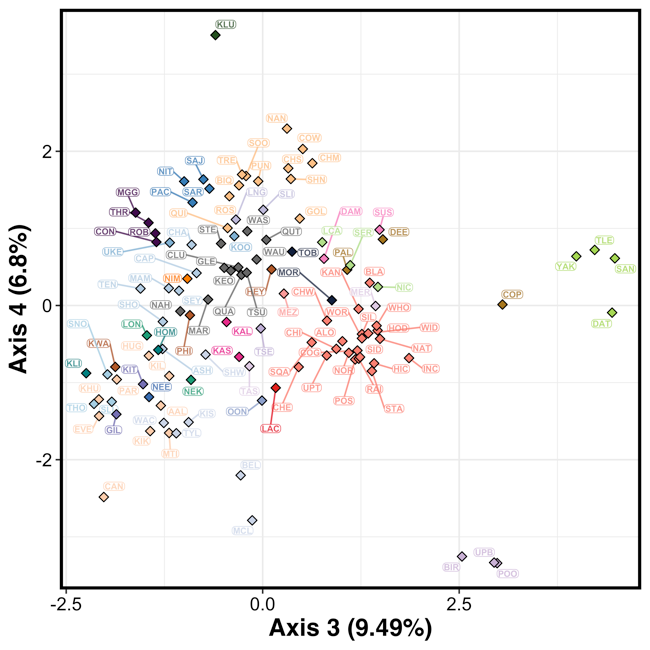

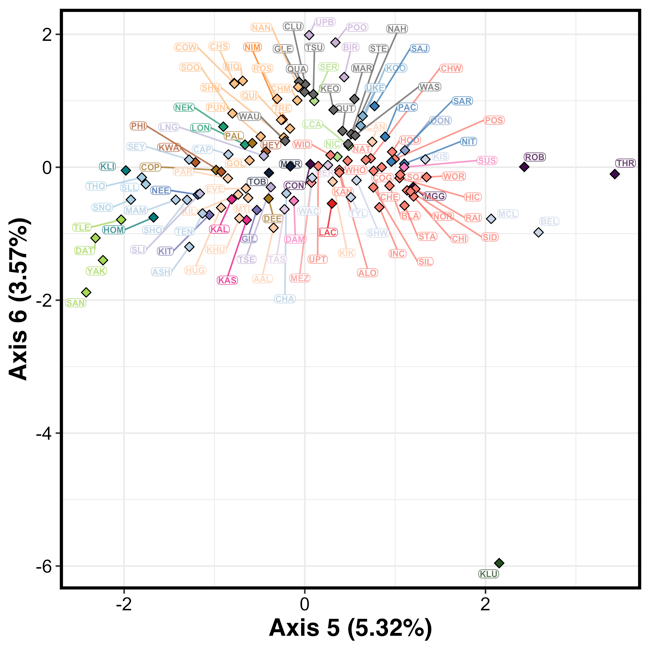


(A) (B) (C)


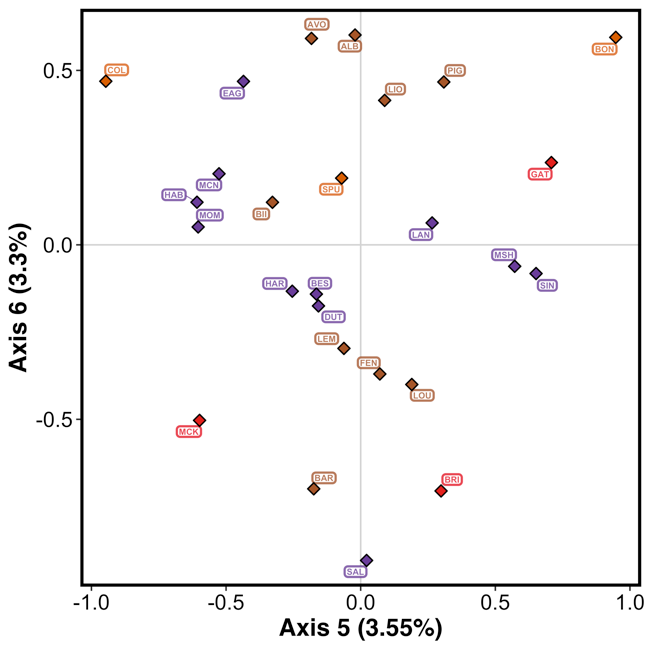

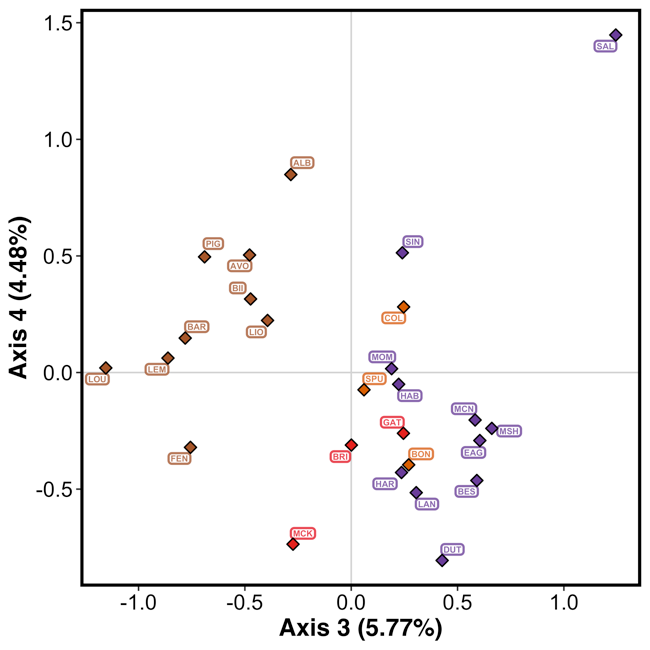

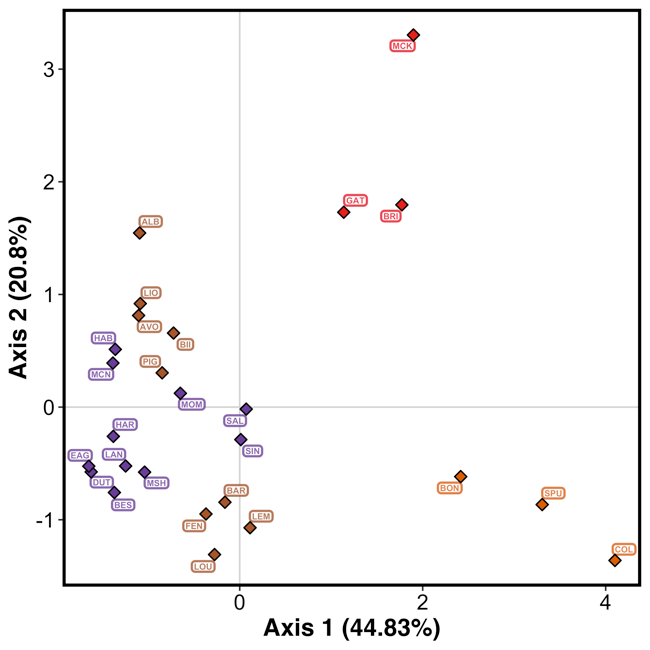


(D) (E) (F)

**Suppl. Fig. 10.** DAPC scatterplots for (A-C) BC and (D-F) Thompson using RDA candidate SNPs. Diamonds represent the centroids of groups (see Suppl. Fig. 13 for plots with points around centroids) coloured according to the CU to which they are designated.


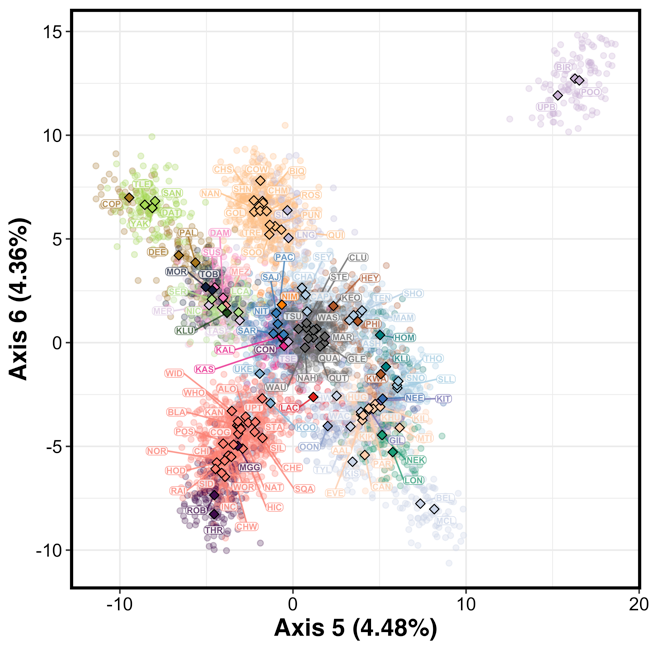

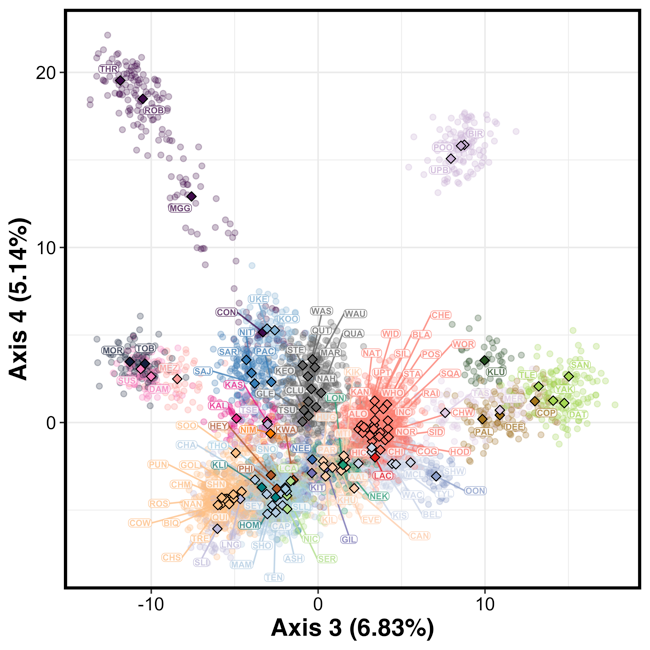

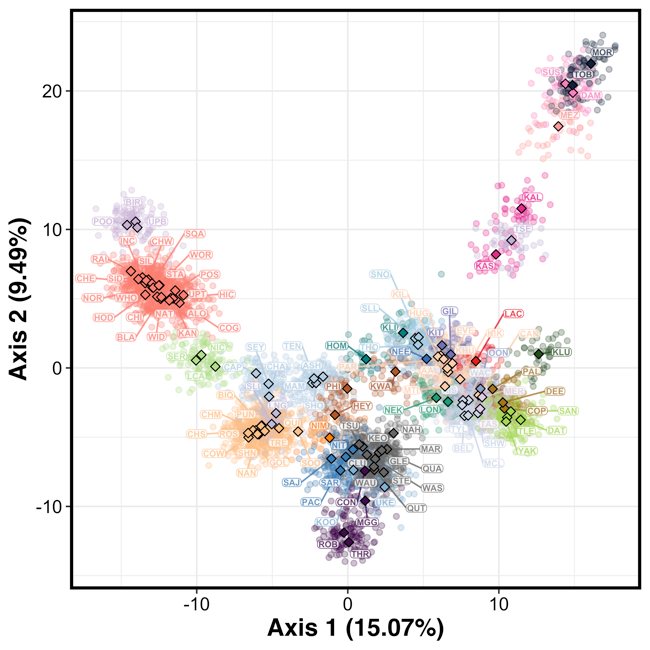
(A) (B) (C)


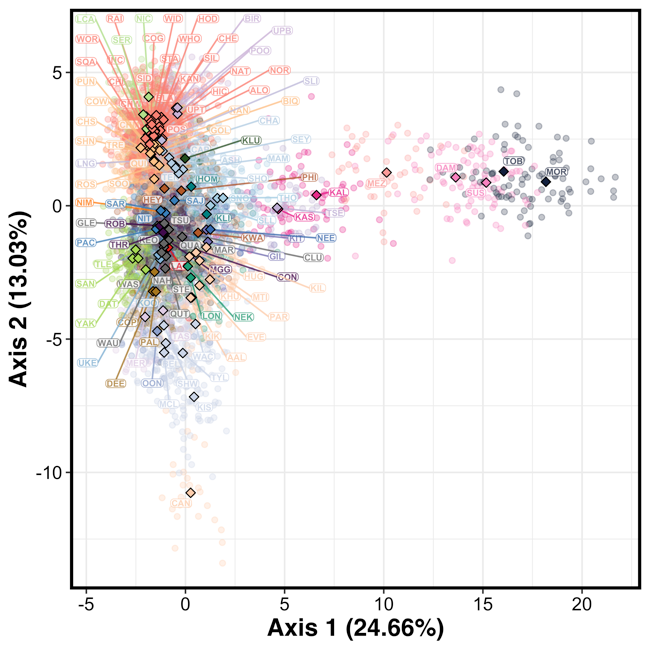

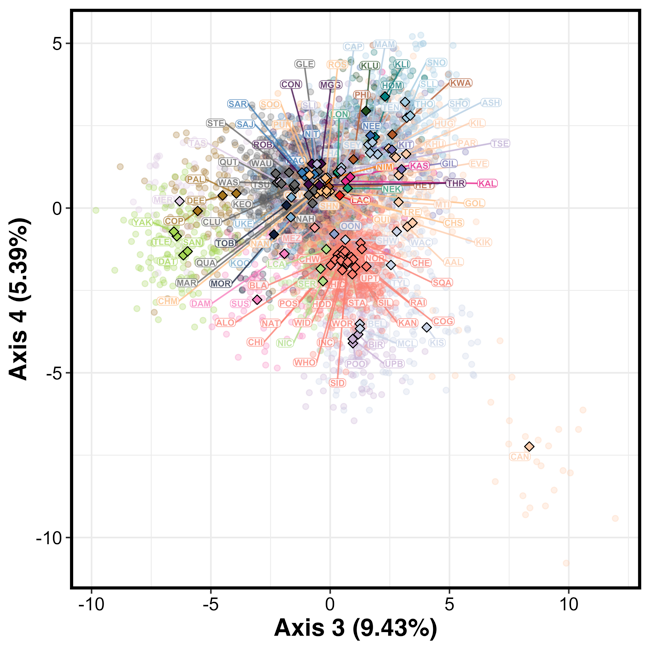

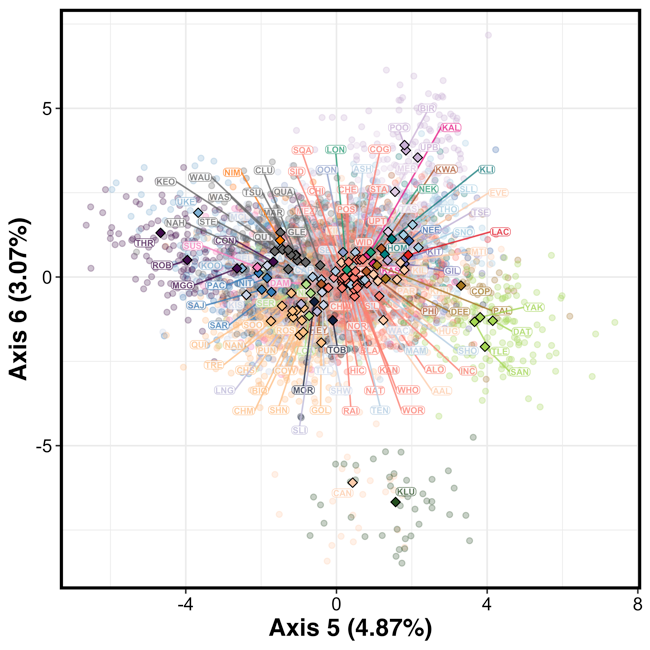


(D) (E) (F)

**Suppl. Fig. 11.** DAPC scatterplots for BC with (A-C) neutral and (D-F) GEA outlier SNPs. Diamonds represent the centroids of groups and points around centroids represent individual samples, coloured according to the CU to which they are designated.


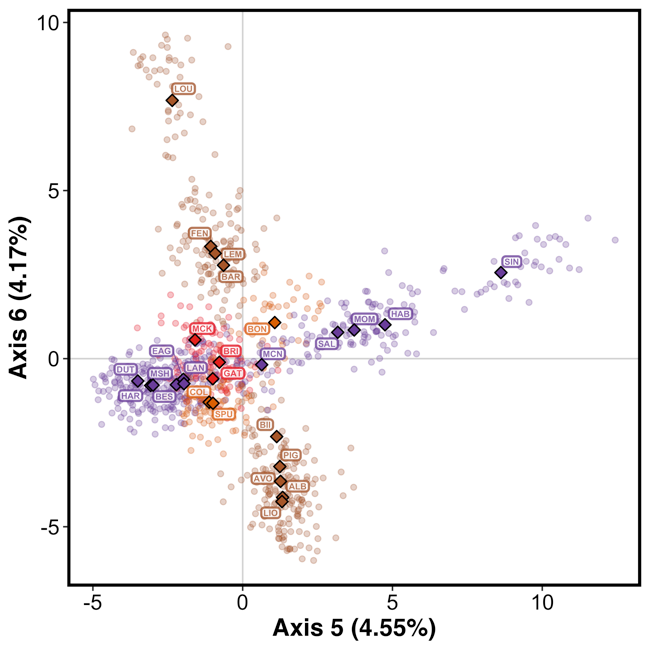

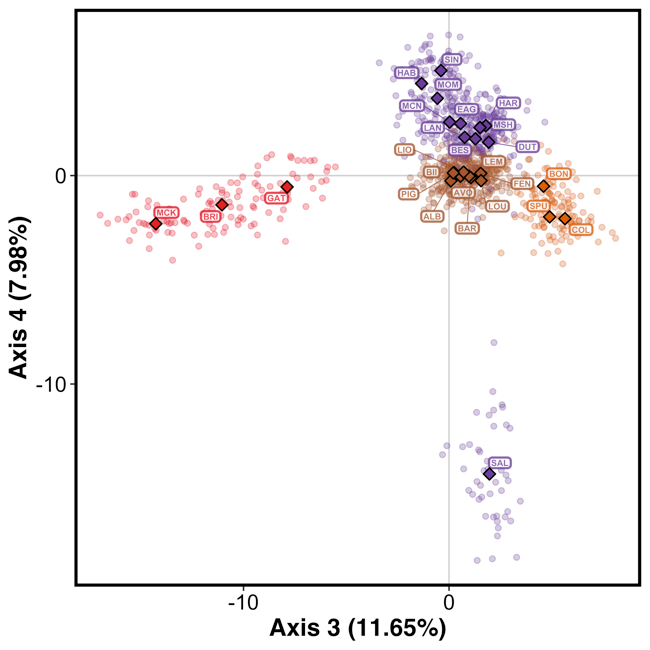

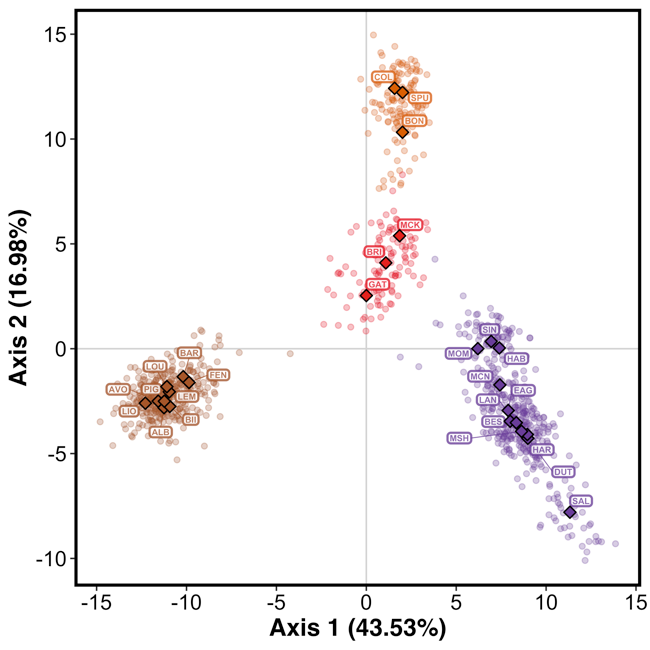


(A) (B) (C)


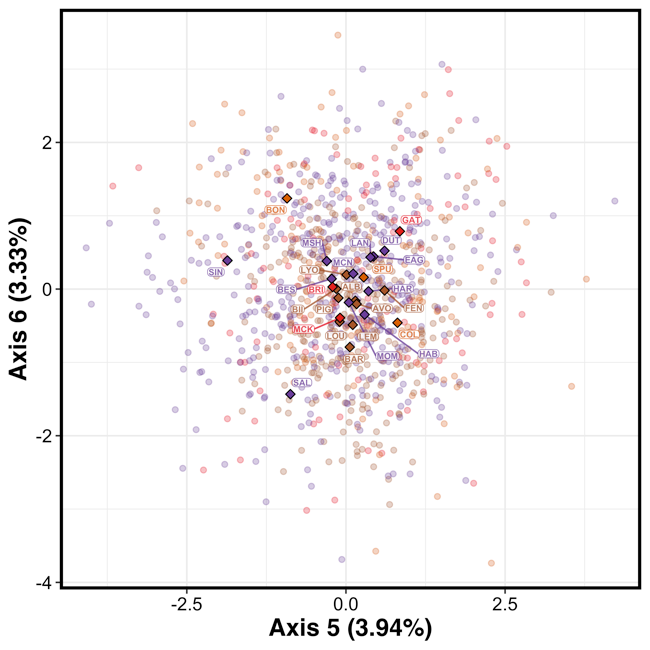

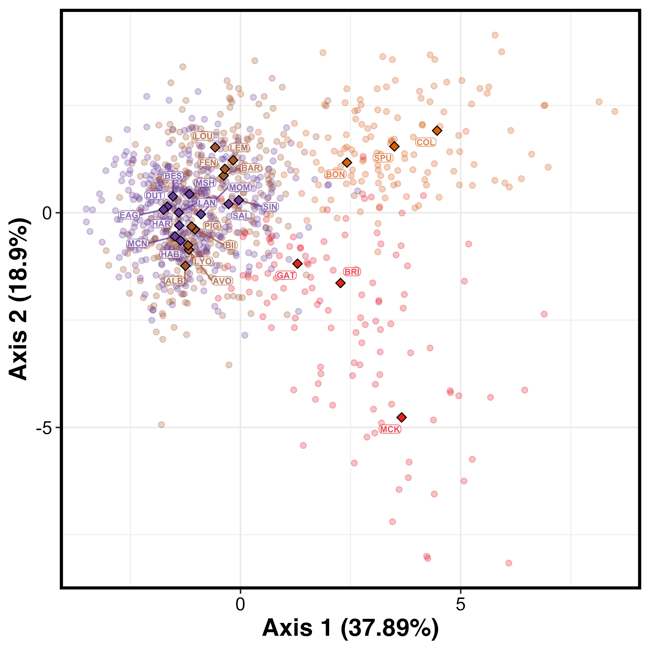

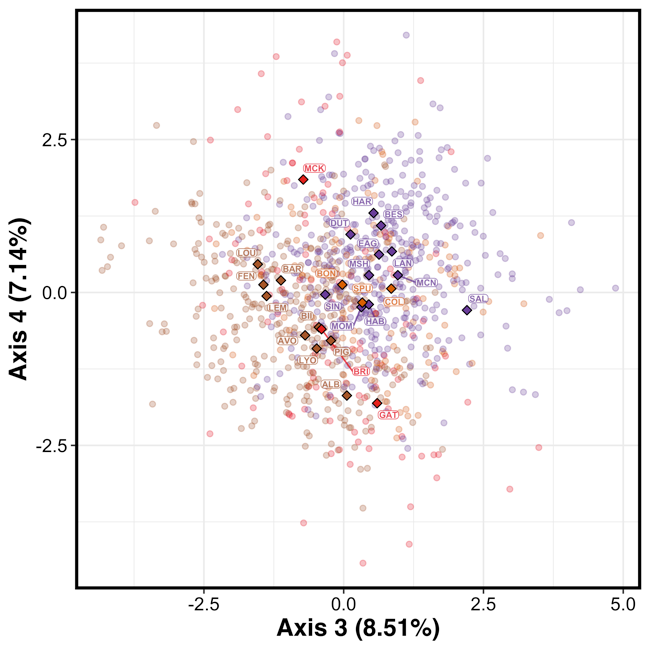


(D) (E) (F)

**Suppl. Fig. 12.** DAPC scatterplots for Thompson with (A-C) neutral and (D-F) GEA outlier SNPs. Diamonds represent the centroids of groups and points around centroids represent individual samples, coloured according to the CU to which they are designated.


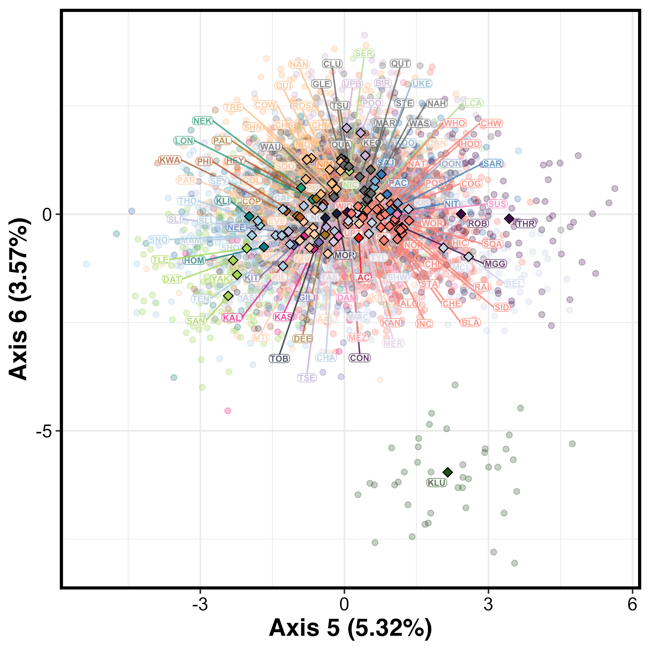

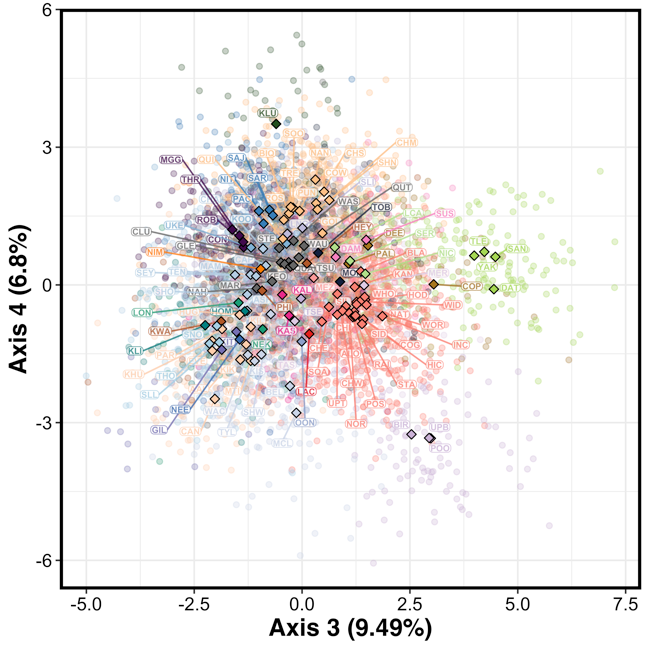

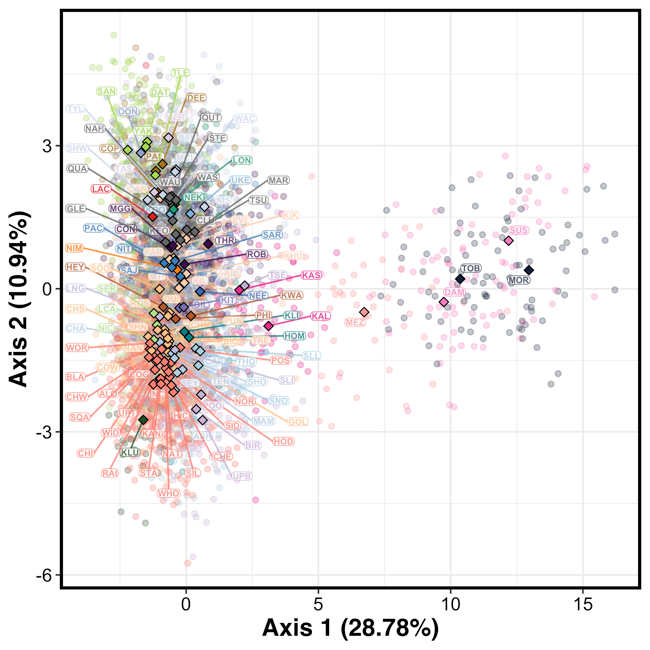
(A) (B) (C)


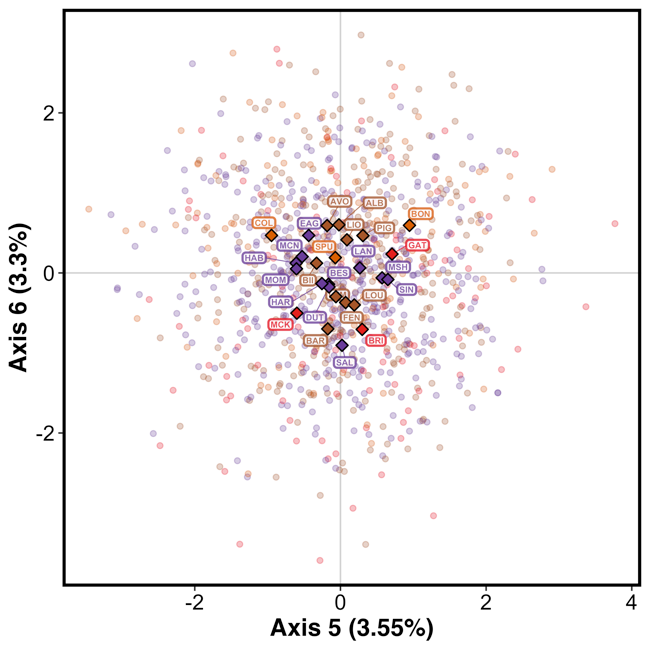

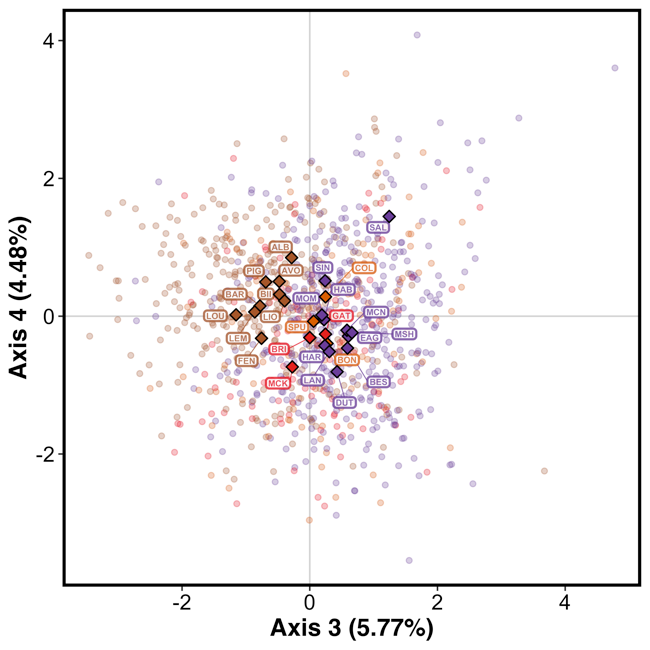

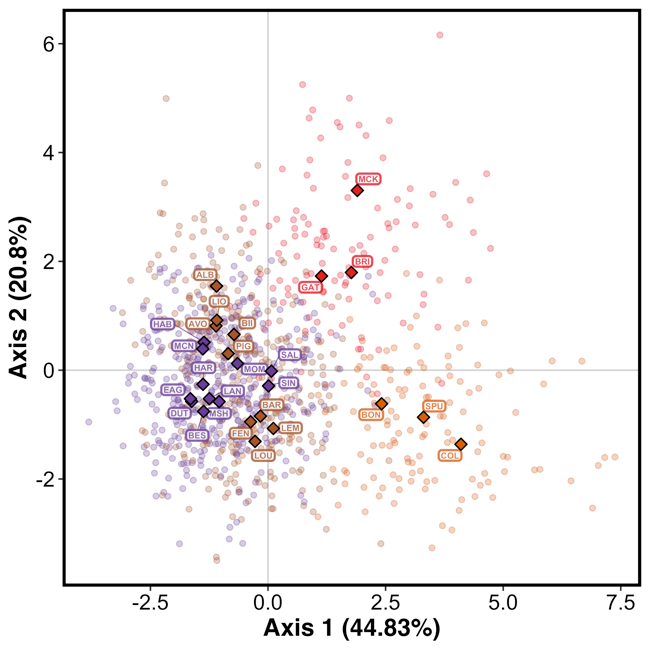


(D) (E) (F)

**Suppl. Fig. 13.** DAPC scatterplots for (A-C) BC and (D-F) Thompson using RDA outlier SNPs. Diamonds represent the centroids of groups and points around centroids represent individual samples, coloured according to the CU to which they are designated.
