# Supplementary material for: Re‐evaluating Coho salmon (Oncorhynchus kisutch) conservation units in Canada using genomic data
Source: Evol Appl. 2022 Oct 18;15(11):1925–44. doi: 10.1111/eva.13489 (PMC9679250; doi:10.1111/eva.13489)
Supplement: Supplementary file 1 — Appendix S1 [file EVA-15-1925-s001.zip › eva13489-sup-0003-TableS5.docx]

The caption for Table S5 should read as follows:

" Full results from redundancy analyses (RDA) performed in each regional group (BC and Thompson): the variance explained by each predictor and axis and significance based on a marginal ANOVA with 1000 permutations, the variance inflation factor (VIF) of each predictor, the eigenvalue and proportion of variance explained by each constrained axis (Prop explained), and the variance and significance of the full model based on an ANOVA with 1000 permutations"

I also noticed that the top panel in that table says, 'coastal BC'. Could that be changed to just 'BC', please. I missed that one, and it would be more consistent with the rest of the tables and the text.
